# Coagulation and complement: Key innate defense participants in a seamless web

**DOI:** 10.3389/fimmu.2022.918775

**Published:** 2022-08-09

**Authors:** Edward L. G. Pryzdial, Alexander Leatherdale, Edward M. Conway

**Affiliations:** ^1^ Centre for Blood Research, Life Sciences Institute, University of British Columbia, Vancouver, BC, Canada; ^2^ Department of Pathology and Laboratory Medicine, University of British Columbia, Vancouver, BC, Canada; ^3^ Canadian Blood Services, Medical Affairs and Innovation, Vancouver, BC, Canada; ^4^ Division of Hematology, Department of Medicine, University of British Columbia, Vancouver, BC, Canada

**Keywords:** complement, coagulation, proteolysis, innate immunity, NETs, contact system

## Abstract

In 1969, Dr. Oscar Ratnoff, a pioneer in delineating the mechanisms by which coagulation is activated and complement is regulated, wrote, “In the study of biological processes, the accumulation of information is often accelerated by a narrow point of view. The fastest way to investigate the body’s defenses against injury is to look individually at such isolated questions as how the blood clots or how complement works. We must constantly remind ourselves that such distinctions are man-made. In life, as in the legal cliché, the devices through which the body protects itself form a seamless web, unwrinkled by our artificialities.” Our aim in this review, is to highlight the critical molecular and cellular interactions between coagulation and complement, and how these two major component proteolytic pathways contribute to the seamless web of innate mechanisms that the body uses to protect itself from injury, invading pathogens and foreign surfaces.

## Introduction

### Historical perspectives

While early sages, including Hippocrates and Aristotle recognized that blood clots rapidly after it leaves the body, it was not until at least the 16th and 17th centuries that scientists determined that clots formed in blood vessels following injury (1). William Hewson showed in the 1770s that blood coagulated and that the so-formed clot was not derived from the cells, but rather from the liquid portion of blood, i.e., the “coagulable lymph” or serum. Studies of the chemical basis for clotting were triggered in the 1870s with the discovery and purification of fibrinogen by the Swedish scientist Olof Hammarsten, and descriptions of prothrombin, “thrombokinase” and calcium as key participants. In 1905, a landmark “classic theory of coagulation” was described by the German physician Paul Morawitz (2), in which coagulation was proposed to occur in two stages, with thrombin generation and fibrinogen coagulation. Thus, the foundation for today’s much more intricate step-wise paradigm was established.

In parallel to this blood clotting system work, in 1888, the American-British bacteriologist, George Nuttall reported that sheep blood was bacteridical for *anthrax bacilli*. This effect was later shown to be negated by preheating the blood to 55°C (3). Thereafter, the German bacteriologist, Hans Buchner named this bactericidal factor “alexine” from the Greek, loosely translated as “protective stuff” (4). Further study by the Belgian scientist, Jules Bordet, determined that the *in vivo* activity of this serum-based, heat-labile alexine was partly dependent on the presence of other heat stable factors. These findings aligned with those of German physician-scientist Nobel laureate, Paul Ehrlich, who had introduced the concept of humoral immunity, and proposed that alexine was indeed complementary to the action of antibodies. And so, in 1899, the term “complement” was coined, relegating alexine to its ancient Greek origins (5).

During the first 30-40 years of the 20th century, there was frenzied activity in both coagulation and complement, with scientists studying these complex systems distinctly, with the goal of establishing mechanistic clarity. Interestingly, in the 1920s and 1930s, Jules Bordet and others published papers claiming that complement and coagulation were related, and indeed, that prothrombin was the “midpiece of complement” (6). This notion was discarded by most, including in 1935 by the famed American chemist, Armand Quick, who made numerous contributions to coagulation, including the prothrombin time test (1).

By the 1930s, a “classical pathway” of the sequential activation of four serum complement fractions C1-C4-C2-C3, had been characterized, triggered by infection-specific immune reactants. This remained the only recognized complement activation pathway until the late 1950s (7), when Louis Pillemer, at Western Reserve University, introduced the notion of an “alternative pathway” with his discovery of properdin (8, 9). The relevance of this alternative pathway is now acknowledged for its central importance in health and disease.

Similarly rapid advances in understanding coagulation and clot dissolution (i.e., fibrinolysis) were made in the mid-1900s, as the conversion of prothrombin to thrombin had been characterized, and factor (F) V to FXIII, to varying extents, were identified, often discovered by analysis of patients with inherited deficiencies and consequent bleeding disorders (1). With the introduction in the 1960s of more powerful protein purification and analytical technologies, and the ability to generate specific polyclonal antibodies, many more components and fragments of the coagulation and complement systems were characterized (10). Interestingly, many of these discoveries in complement and coagulation occurred simultaneously - not infrequently in adjacent laboratories. Indeed, Louis Pillemer and Oscar Ratnoff were colleagues at Western Reserve University and famously collaborated on multiple works that bridged these disciplines (11) and led to the wisdom that our “body protects itself [by] a seamless web, unwrinkled by our artificialities” (12).

Over the past several decades, it has become widely accepted that complement and coagulation must be viewed as inextricably intertwined. The biochemical pathways intersect and impact the other’s endpoints. The cells and molecular pathways with which each communicates are shared. The finely tune balance of initiating factors, and the site and timing of activation, amplification and resolution of coagulation and complement are most often overlapping, and most certainly coordinated *via* complex feedback mechanisms. Most notably, genetic studies in conjunction with sensitive biochemical assays and an improved understanding of the interplay between these pathways and their common cellular and molecular partners, have revealed mutations in complement regulatory factors. These cause excess complement activation, resulting in feedback-mediated hyper-coagulation and hyper-inflammation, leading to tissue damage and organ failure. Thus, introduction of remarkably effective anti-complement drugs for paroxysmal nocturnal hemoglobinuria (PNH; see **Table 1** for a list of abbreviations) and atypical hemolytic uremic syndrome (aHUS) (13, 14), two devastating complement-mediated thrombotic disorders, underlines the value of bringing these two biochemical proteolytic pathways together in the clinic.

**Table 1:** Table of Abbreviations.

ADAMTS13	a disintegrin and metalloproteinase with a thrombospondin type 1 motif, member 13
aHUS	atypical hemolytic uremic syndrome
AP	alternative pathway
APAS	antiphospholipid antibody syndrome
aPL	anionic phospholipid (e.g., phosphatidyl serine)
AT	anti-thrombin
BK	bradykinin
C1-INH	C1 inhibitor
C3aR, C5aR, C5aL2	receptors for C3a and C5a
CP	classical pathway
CPB2	carboxypeptidase B2
CS	contact system
CsA	chondroitin sulfate A
DAMP	damage/danger associated molecular pattern
FB	factor B
FD	factor D
FH	factor H
HITT	heparin induced thrombocytopenia and thrombosis
HK	high molecular weight kininogen
LP	lectin pathway
MAC	membrane attack complex (C5b-9)
MASP	MBL-associated serine protease
MBL	mannose binding lectin
MMP	matrix metalloprotease
NETs	neutrophil extracellular traps
PAI-1	plasminogen activator inhibitor-1
PAMP	pathogen associated molecular pattern
PAR	protease activated receptor
PC, APC	protein C, activated PC
PF4	platelet factor 4
Pg	plasminogen
PGI	prostacyclin
PK, PKa	plasma prekallikrein, plasma kallikrein
PNH	paroxysmal nocturnal hemoglobinuria
PRM	pathogen recognition molecule
prothrombinase	Factor Va/Factor Xa (FVa/FXa) enzymatic complex
PSGL-1	P-selectin glycoprotein 1
SUSD4	sushi domain-containing protein 4
TAFI	thrombin activatable fibrinolysis inhibitor, activated TAFI
tenase	TF/Factor VIIa (TF/FVIIa) enzymatic complex
TF	tissue factor
TFPI	tissue factor pathway inhibitor
tPA	tissue type plasminogen activator
ULIC	ultra large immune complexes
VWF, ULVWF	von Willebrand Factor, ultra large multimeric VWF

In spite of the tremendous increase in our understanding of the molecular mechanisms by which coagulation and complement pathways intersect, there is much more to be learned. In this report, we review some of the more recent and prominent discoveries of how these complex, primarily blood-borne proteolytic cascades, interact and indeed, how they interface with other critical pathways involved in innate defense. We begin with brief descriptions of each of the pathways (the reader is referred to other publications for in-depth reviews), and then highlight some of the key, potentially relevant cellular and molecular links that tie them into a cohesive, albeit complex unit. Throughout this discussion, we are reminded of Dr. Ratnoff’s caution (12), that the distinction of these pathways is man-made, based partly on the limitations of *in vitro* test-tube analyses; and in reality, complement and coagulation are just two of several interacting participants in a seamless web of innate pathways, designed to effectively and efficiently protect the host.

## The coagulation system

### Coagulation overview

Cardio-cerebrovascular diseases and thromboembolic disorders have been the leading causes of death globally for >100 years (15). The blood coagulation system has thus long been at the forefront of scientific study [for reviews (16–19)]. The system features a series of reactions in which a specific injury or stimulus triggers a cascade of limited proteolysis, whereby inactive proteins (zymogens) are converted sequentially to their respective proteases, amplified at each step to ultimately generate a fibrin clot (1, 20). Coagulation-related protease systems are tightly regulated and delicately balanced to maintain vascular homeostasis under a wide range of pathophysiologic stimuli (21). Thus, while the system is continuously active, sequential proteolysis of zymogen to active protease, and protease activity on respective substrates are held in check at each step under healthy conditions, by an array of natural anticoagulant mechanisms (22), disruption of which results in a thrombotic diathesis. These coagulation protease components and regulators not only modulate the hemostatic-thrombotic response, but also participate in other fundamental biologic processes including innate and adaptive immunity, inflammation, cell proliferation, wound healing, and cancer. It follows that dysregulation of the coagulation system may have far-reaching effects.

### Coagulation initiation: Platelets, anionic phospholipid and tissue factor

Blood coagulation is initiated at sites where the vascular endothelial cell monolayer is damaged. This exposes the subendothelial stratum which contains molecules, such as collagen and von Willebrand Factor (VWF), to plasma and circulating platelets which adhere, activate and aggregate (23), thereby forming the primary vascular seal. Clot formation stabilizes the platelet plug, which alone is insufficient to withstand the shear forces of blood flow. The platelet response to vascular damage, features regulated flipping of anionic phospholipids (aPL: e.g. phosphatidylserine) from the inner to the outer cell membrane bilayer leaflet (19, 24) and the release of granule contents (25), both of which are relevant to the initiation and amplification of coagulation. Platelets release important hemostatic constituents into the circulation from α- and dense-granules (26), including, for example, platelet activating factor, platelet factor 4 (PF4), P-selectin, adenosine diphosphate and polyphosphate. These elicit local cell stimulatory effects, recruit and activate neutrophils and monocytes, and may promote further accessibility of aPL, an essential cofactor for assembly of all coagulation cofactor/enzyme protein complexes.

**Figure 1:**
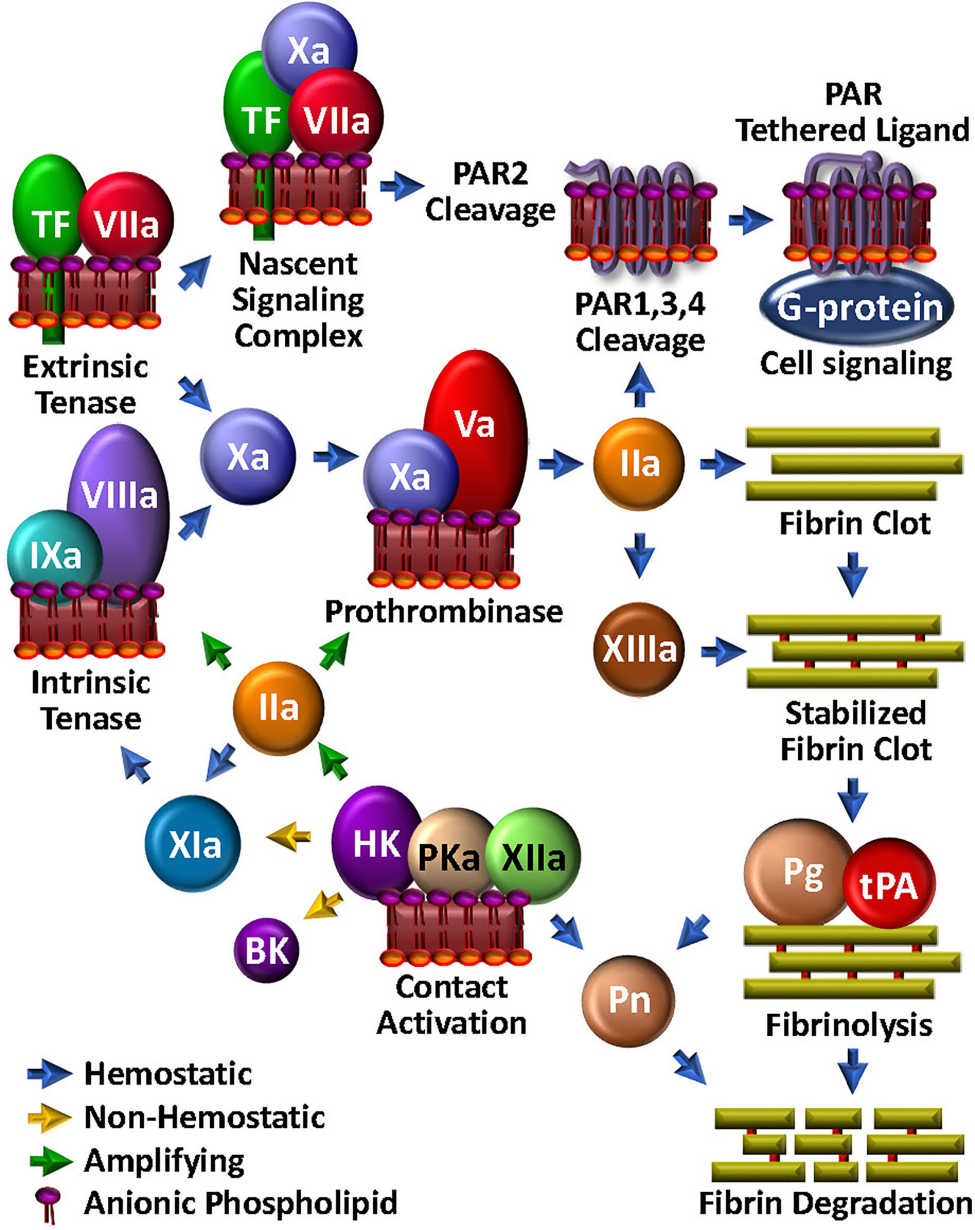
Coagulation Unwebbed. Upon vascular damage, hemostatic coagulation is initiated by exposure of TF and assembly of the extrinsic tenase, leading to prothrombinase and ultimate thrombin (IIa) production, which is responsible for direct fibrin clot formation and feedback amplification involving the intrinsic tenase. The nascent TF/FVIIa/FXa complex and thrombin facilitate PAR-mediated cell modulation. FXIIIa crosslink-stabilizes the clot. Initiation and amplification of coagulation may be facilitated by the so-called intrinsic coagulation/contact pathway. Clot degradation and solubilization is facilitated by the fibrinolysis pathway through tPA-mediated plasminogen (Pg) activation to plasmin (Pn), which can be enabled by kallikrein (PKa).

The essential physiologic trigger of coagulation is the transbilayer receptor, tissue factor (TF). Normally present throughout the vasculature in a subendothelial pool, TF becomes available, i.e., it is decrypted and activated, upon vascular damage (27, 28) (**Figure 1**). Binding of the zymogen, factor (F)VII to TF, drives a conformational transition that enables autoproteolytic activation to FVIIa (29). TF then accelerates FVIIa-mediated activation of FX by ~100,000-fold (30, 31) to form the first FXa molecules. TF/FVIIa constitutes the extrinsic tenase, which can also initiate activation of FIX (32) and FVIII (33). FIXa and FVIIIa are the protease and cofactor within the intrinsic tenase, respectively. Like all coagulation protease complexes, tenase assembly and function are Ca^2+^- and aPL-dependent. Thus, together, TF and aPL are integral for initiation and localization of the procoagulant response to vascular damage. Particularly relevant to our discussion, TF is also located on endothelial cells and activated leukocytes, thereby well-positioned for mediating critical roles not only within, but also beyond coagulation.

### Feedback amplification by thrombin

Upon generation of sufficient FXa to overcome endogenous circulating anticoagulants, such as tissue factor pathway inhibitor (TFPI) (34) or antithrombin (AT) (35), FXa then activates its cofactor, FV to FVa (36) (**Figure 1**). Assembly of the Ca^2+^-dependent FVa/FXa prothrombinase complex on an aPL-containing membrane results in cleavage of prothrombin to generate the potent serine protease thrombin (37) which in turn triggers polymerization of soluble fibrinogen by proteolytic conversion to fibrin cross-linked by thrombin-activated FXIII to yield a stable clot.

Thrombin recognizes several protein substrates that contribute to its own generation and explosive feedback amplification (16). Thus, it activates FV and FVIII, and converts FXI to FXIa, the latter which contributes to generating the intrinsic tenase activity by further activating FIX. Combined with the newly available FVa, up-regulated FVIIIa/FIXa tenase activity tremendously augments downstream prothrombinase assembly and the ensuing production of thrombin.

Small amounts of TF-triggered coagulation proteases, including thrombin and FXa, also elicit a wide range of thromboinflammatory effects on neighbouring platelets, neutrophils and endothelial cells *via* direct activation of cell surface expressed PARs (38) (**Figure 1**). These are a group of four homologous receptors (PAR1-4) that are expressed on numerous cell types (39). PAR1 is the high affinity receptor for thrombin (40, 41), but may be cleaved by several proteases. PAR2 is expressed on leukocytes and endothelial cells, and rather than thrombin, is activated by FXa, particularly in the context of the TF/FVIIa/FXa complex. Through its direct cell agonist effects on PAR2 and indirect effects on PAR1 *via* thrombin generation, TF is therefore well integrated in the thromboinflammation web, with key roles in bridging coagulation, complement and inflammation (42).

### Fibrinolysis: Plasmin-mediated clot dissolution and cell modulation

Once the fibrin/platelet clot has sealed the damaged vasculature, fibrinolysis is initiated to restore normal blood flow and effect healing by assembly of the protease/substrate tissue-type plasminogen activator (tPA)/plasminogen (Pg) complex, directly on the remaining fibrin (**Figure 1**). Fibrin serves to localize and orient the enzyme/substrate complex for efficient proteolysis of the zymogen Pg, to its respective serine protease, plasmin. With generation and activity of plasmin tightly regulated by plasminogen activator inhibitor (PAI)-1 and alpha-2-antiplasmin, plasmin can effectively solubilize the clot and trigger cellular events that facilitate healing. Indeed, the substrate specificity of plasmin is broad (43). For example, like thrombin, plasmin cleaves PAR1, and thus participates in platelet activation (44), macrophage release of proinflammatory cytokines (45) and expression of TF (46) on monocytes. Plasmin can also activate PAR2, thereby modulating endothelial function (47). Finally, plasmin activates several matrix metalloproteases (48–50), which in turn, may impact on inflammation, hemostasis and tissue remodeling (51). Not surprisingly, plasmin has direct effects on complement activation, which will be further discussed below.

## The complement system

### Complement overview

A major blood-based proteolytic system, complement is orchestrated to innately respond as a first-line protector of the host from invading pathogens and damaged cells (52, 53). Like coagulation, the complement system is delicately balanced, tightly regulated and highly versatile, integrated with other innate and adaptive response systems, and linked to physiologic systems that prevent bleeding, and effect cell proliferation, tissue regeneration and healing (54). Mechanisms of generation and regulation of key proteolytic enzymes in complement versus coagulation, while similarly dependent on key ions and surface interactions, are distinct. Under healthy conditions, the complement system functions in a low activity-level surveillance mode. Like coagulation, it rapidly escalates to address the insult, in a highly localized and temporal manner. Thus, when danger signals to the host have been sufficiently averted, complement system activity defervesces, leaving biologically active proteolytic by-products that participate in recruiting inflammatory cells and adaptive immune responses, to ensure healing and a return to homeostasis.

### Converging three complement initiating pathways

**Figure 2:**
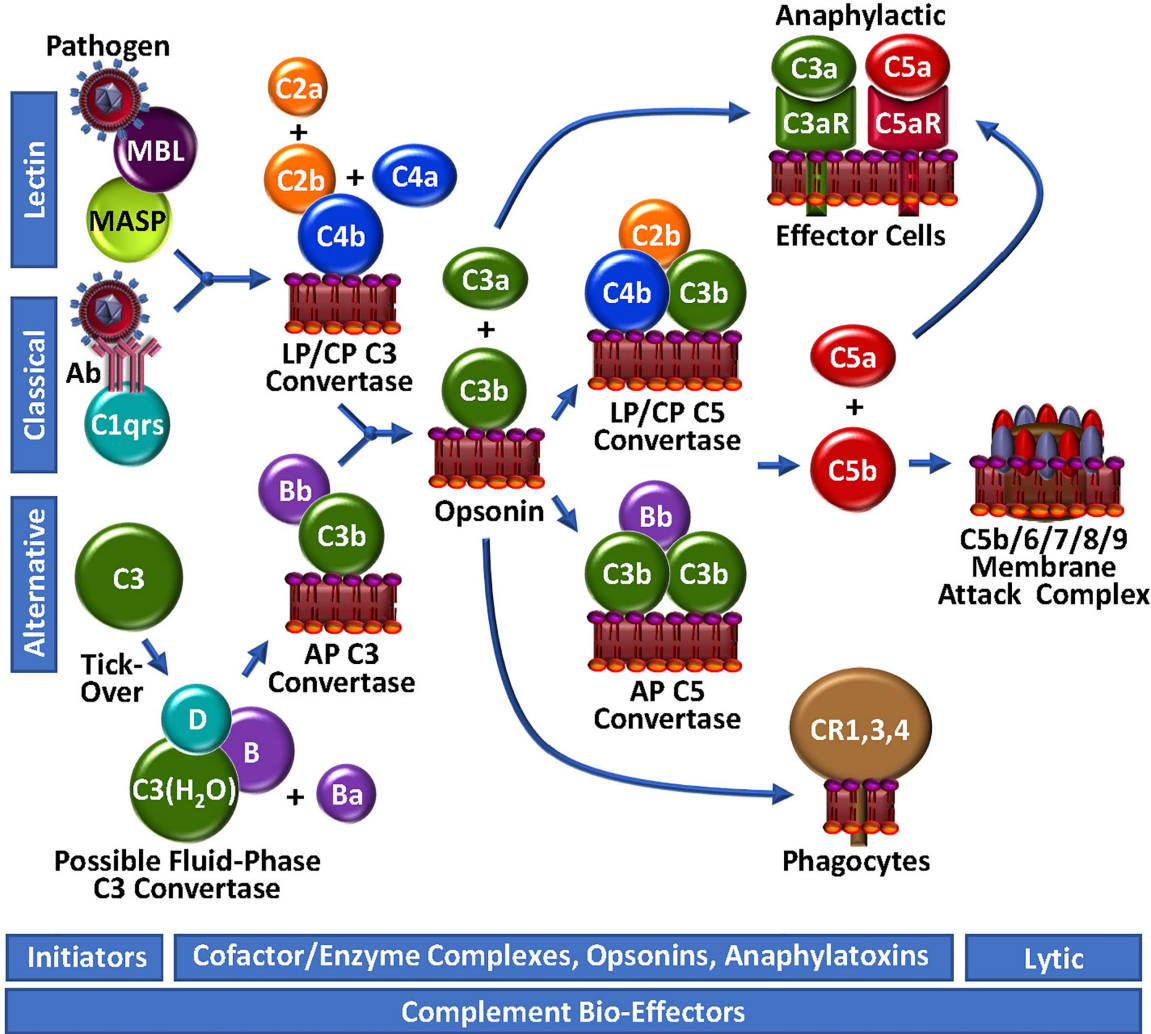
Complement Unwebbed. The LP and CP are initiated by contact with a foreign particle or damaged cells, whereupon C4b is surface-deposited in complex with C2b, forming the LP/CP C3 convertase. The AP is continuously surveying the circulation for foreign bodies by spontaneous thio-ester hydrolysis and possible formation of a highly unstable fluid-phase C3 convertase. Either the LP/CP or AP C3 convertase may result in deposition of surface C3b and generation of respective C5 convertases. C5b production triggers the assembly of the lytic membrane attack complex by the addition of C6, C7, C8 and multiple C9 molecules. Surface-bound C4b and C3b are opsonic, as are degradation fragments of C3b (not depicted), which associate with complement receptors (CR1,3,4). C3a and C5a are anaphylactic, associating with C3aR and C5aR, respectively.

Complement initiation is traditionally viewed as proceeding *via* three pathways – classical (CP), lectin (LP) and alternative (AP) - all converging with the formation of C3 convertases that proteolyse C3 into cofactor, C3b, with release of the anaphylatoxin, C3a (**Figure 2**). Initiation of complement *via* the CP and the LP requires that complement components participate as pathogen recognition molecules (PRM) by direct or antibody-facilitated detection of a wide range of pathogen and damage/danger associated molecular patterns (PAMPs and DAMPs) perceived as being foreign. These include, for example, DNA, RNA, modified lipids, oligosaccharides, histones, heat shock proteins, formyl peptides, plasma membrane constituents, and/or extracellular matrix proteins (55), any of which may be exposed/released upon pathogen invasion and/or with cellular damage.

The initiator of CP activation is C1q, a multivalent PRM that continuously surveys blood in complex with a tetramer of the zymogen forms of the serine proteases C1r and C1s (C1qr_2_s_2_). With an infection or injury, C1q activity is triggered by the Fc regions of specific “complement-fixing” antibodies that are bound to neo-antigens and/or microbial surfaces (**Figure 2**). C1q can also recognize other targets, including C-reactive protein, modified lipids and carbohydrates. With binding of C1q to its target, the zymogen C1r undergoes a Ca^2+^-dependent conformational change to a serine protease (56), which in turn, cleaves/activates its neighbouring zymogen, C1s, to its serine protease form (57). C1s proteolyses C4 to generate two fragments: C4a is a weak anaphylatoxin, while C4b contains a reactive thioester, allowing it to covalently bind to the surfaces of nearby damaged cells/pathogens. The immobilized C4b provides a binding site for C2, which is subsequently cleaved by C1s into C2b and C2a. While C2a is liberated in a soluble form, C2b complexes with the cofactor C4b, yielding the CP C3 convertase, C4b/2b, which proteolytically catalyzes the generation of C3b and C3a from C3, thus propagating complement.

The LP follows a similar course as the CP to generate C4b/2b (58), but the triggering events are distinct. LP PRMs comprise mannose binding lectin (MBL), ficolins or collectin-11, which may individually circulate in complex with MBL-associated zymogens of serine proteases (MASP), MASP1/MASP3 and MASP2. These multi-molecular complexes specifically bind to sugars or N-acetylated groups on micro-organisms. MASP1 then autoactivates in a Ca^2+^-dependent manner, allowing it to cleave C2 and MASP2. MASP2 also autoactivates and cleaves both C2 and C4, resulting in the formation of the C4b/2b LP C3 convertase, which is identical to the CP C3 convertase (59).

**Figure 3:**
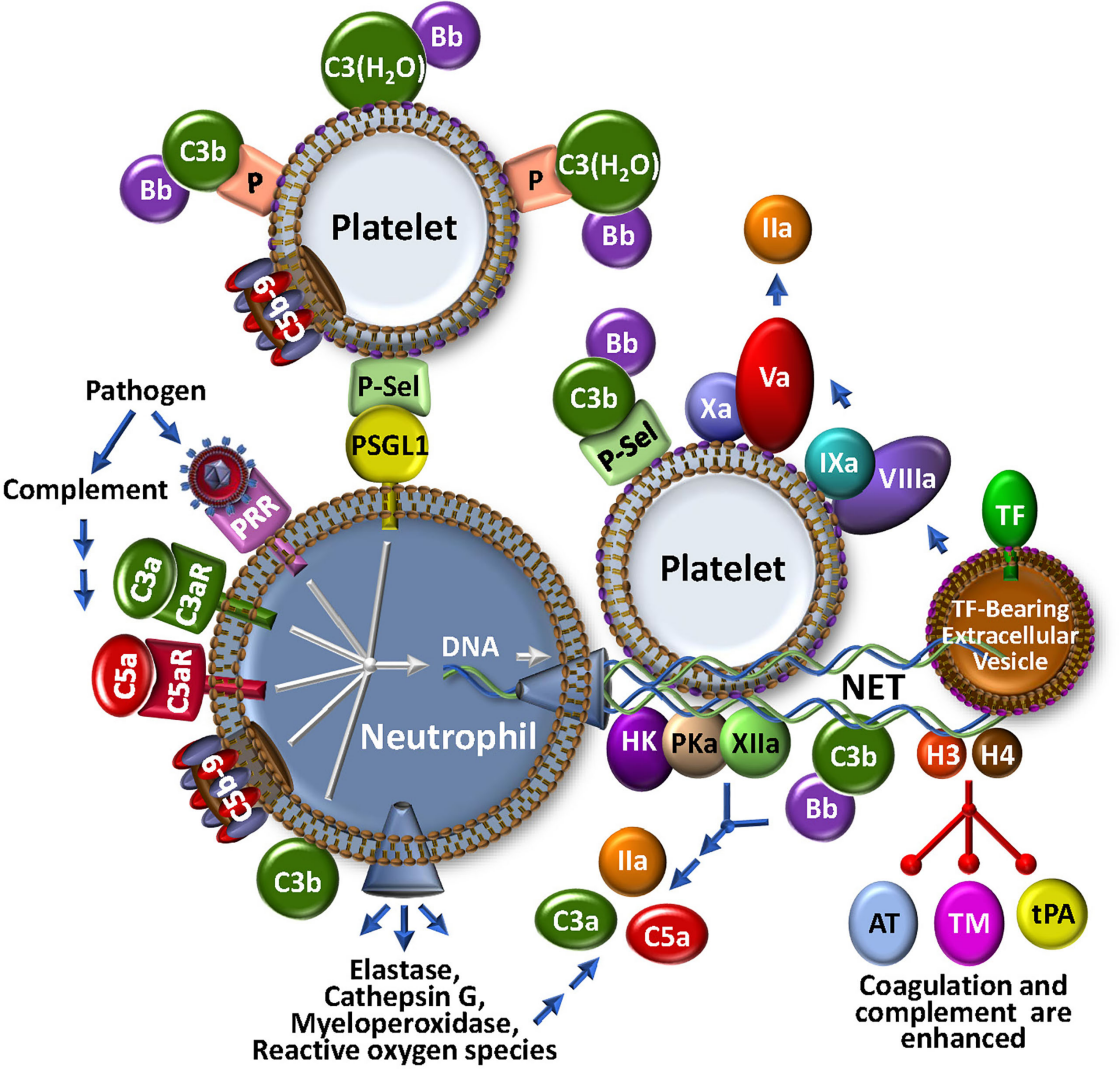
The NETosis-Coagulation-Complement Web. Neutrophils release NETs after binding of foreign particles to pathogen recognition receptors (PRR), engagement of complement anaphylatoxic or opsonic species, or association of P-selectin on activated platelets with P-selectin glycoprotein 1 (PSGL1). Stimulated neutrophils release numerous bioeffectors including neutrophil elastase, cathepsin G, myeloperoxidase and reactive oxygen species, which may affect production of coagulation and complement bioeffectors directly or indirectly through cell modulation. Release of DNA-based NETs from neutrophils, provides a complex matrix for interactions with stimulated platelets and localizes TF activity, possibly by trapping extracellular vesicles. Both coagulation and complement pathways are further propagated by direct protein factor association with NETs through the contact and alternative pathways, respectively. Multistep crosstalk between these pathways results in further generation of thrombin, C3a and C5a with potential for biological consequence. Histones, H3 and H4, are major protein constituents of NETs and enhance coagulation and complement by inhibiting the regulators, antithrombin (AT) and thrombomodulin (TM), and stabilize the clot by attenuating tPA-mediated fibrinolysis. The activated platelet surface is typically regarded as procoagulant because it provides anionic phospholipid (purple polar head groups), but platelet-bound P-selectin and properdin (P) also stabilize AP C3 convertase assembly *via* association with C3b and C3(H_2_O), respectively. The platelet surface may also associate directly with C3(H_2_0) toward complement activation.

While the CP and LP apparently require an “on” signal, the AP has historically been viewed as being constitutively active (**Figure 2**). This is analogous to the coagulation system, which is also in a continuous “ready” state, always minimally generating low levels of FVIIa and thrombin. In the AP of complement, this is reportedly achieved *via* the so-called “tick-over” mechanism to describe spontaneous thioester hydrolysis that circulating C3 undergoes, transforming it into C3(H_2_O) (60). In more recent studies, this theory has been adapted/questioned with the suggestion that AP activation of C3 to C3(H_2_O) may require contact - or at least may be initiated and accelerated by contact - with one or more biological or artificial surfaces, such as lipids/lipid complexes, gas interfaces or biomaterials (61, 62). A continuous source of C3(H_2_O) in the plasma may also be activated platelets (**Figure 3**) which transform C3 to C3(H_2_O) and stabilize other AP convertases (61, 63). Regardless of the mode, once it is generated, C3(H_2_O) exposes a Mg^2+^-dependent binding site for circulating zymogen factor B (FB) (64). Factor D (FD) [also known as adipsin (65)], a low abundance serine protease in serum that is secreted as a pro-enzyme by adipocytes, endothelial cells and monocytes (65, 66), may proteolyse its only known substrate, FB, in complex with C3(H_2_O), liberating a soluble activation fragment Ba, and a larger Bb fragment. FD is activated by MASP1 and/or MASP3 (67–69), and interestingly, also possibly by thrombin (68, 69), thereby establishing a direct connection between the LP and the AP, as well as the coagulation system. The FD-generated Bb contains an active serine protease domain and remains bound to C3(H_2_O), stabilized by properdin (70, 71), yielding C3(H_2_O)/Bb, a short-lived relatively unstable fluid-phase C3 convertase, that can cleave C3 to C3a and C3b. The reactive thioester exposed by C3b allows covalent deposition onto an amine or hydroxyl group on a nearby surface. In the absence of potent negative regulators, including for example, factor H (FH), CR1 and CD55, circulating FB binds to the immobilized C3b, and following FD cleavage of FB, results in the assembly of C3b/Bb, the AP C3 convertase that can be stabilized by properdin (70), followed by subsequent downstream complement activation.

### Complement amplification: C3b generation

The generation of C3b by cell-bound C4b/2b or C3b/Bb C3 convertases, converges the 3 initiating branches of complement (**Figure 2**). At this point, AP-mediated amplification may occur as more C3b is locally deposited, and more C3b/Bb complex is rapidly formed. This represents an unstable and transient non-covalent assembly, with ensuing rapid decay that is normally accelerated by control proteins, such as FH and CD55. However, unchecked by reduced functional expression of one or more of these negative regulators, the additional C3b molecules bind to the C3 convertases to form respectively, the CP/LP and AP C5 convertases, C4b/2b/C3b and C3b/Bb/C3b, resulting in a shift in substrate specificity favoring cleavage of C5 over C3. Further amplification is facilitated by a complex between C2b and a covalent C4b dimer, likely produced at low-levels, but uniquely not involving the participation of C3b or C3(H_2_O) in the enzymatic complex to yield C3 and C5 convertase activity (72).

### The terminal pathway and the membrane attack complex

C5 convertases cleave C5 into C5b, liberating the potent biologically active anaphylatoxin, C5a (**Figure 2**). Generation of C5b marks the start of the terminal pathway, which spontaneously proceeds in an ion-independent manner, with rapid formation of a stable C5b,6 complex. Subsequent binding of C7 yielding the C5b-7 complex, provides first attachment of the complex to the outer leaflet of a target membrane. Addition of C8 and polymerization of ~18 C9 monomers, completes assembly of the C5b-9 pore-like, lytic membrane attack complex (MAC).

### Bioactive complement activation products: ‘Small but mighty’ fragments

In the course of complement activation and regulation, several proteolytic by-products are generated, most of which are biologically relevant, not only for assembly of the convertases, but also in mediating further activation/amplification of complement, recruiting inflammatory cells and adaptive immunity *via* opsonization (e.g., C3- and C4-derived fragments: C3b, iC3b, C3c, C3d/g, iC4b, C4d) and triggering effects on other biological systems (e.g., anaphylatoxins C5a, C3a, C4a), including coagulation. Indeed, C3a and C5a interact with their cognate G-coupled receptors (C3aR for C3a; C5aR and C5L2 for C5a), thereby mediating diverse activities that are context-dependent, modulated by expression levels of their receptors, and local factors that control their clearance. These small but mighty fragments have profound cell modulatory effects on the immune system that, as will be discussed, spill-over into diverse responses in coagulation and beyond (73–76).

## Complement and coagulation and the seamless web

### Neutrophils and neutrophil extracellular traps: the web’s NETs

In parallel with recruitment and adhesion of platelets and monocytes to damaged vascular endothelium at the outset of a localized infection and/or vascular inflammatory insult, neutrophils are early and key participants in thromboinflammation. Neutrophils innately respond to many stimuli, including PAMPs, DAMPs, N-formyl peptides, as well as - importantly - complement-generated anaphylatoxins C3a and C5a (**Figure 3**). Neutrophils are short-lived effector cells that are phagocytic, and can release multiple proteolytic enzymes (e.g., elastase, myeloperoxidase, metalloproteases, cathepsins), reactive oxygen species, cytokines and chemokines, and PF4. These may impact profoundly on thromboinflammation, for example, by further activating platelets, activating coagulation factors V, VIII and X, and complement component C5 through specific proteolysis, and inactivating natural anti-coagulant/anti-complement factors TFPI, thrombomodulin and AT (77). Stimulated neutrophils are consequently an important fulcrum for localized regulatory complement-coagulation mechanisms. It follows that major research efforts are being directed to better understand the molecules that effect neutrophil priming, activation and release. Among others, these include the C5a/C5aR axis and neutrophil chemokines such as CXCL8, neutrophil elastase and CXCR2 (78–80), some of which are being explored as therapeutic targets.

Activated neutrophils can also release neutrophil extracellular traps (NETs) (**Figure 3**), a process referred to as NETosis. NETs are web-like structures that are secreted during a specialized type of cell death where the cell remains intact and retains certain biological function (81, 82). NETs can also be released by activated eosinophils, basophils and monocytes. NETs trap and kill bacteria and invading pathogens, and provide a scaffold for aggregating platelets and red blood cells, contributing to thrombosis and thromboinflammation (83, 84). They are provoked by various stimuli, including bacteria and viruses, activated platelets, hypoxia, reactive oxygen species and cytokines (81, 85). Discharge of NETs by neutrophils can also be triggered by C3a and C5a *via* interactions with their receptors, C3aR and C5aR, respectively, by C5b-9 (see below), as well as following opsonization with C3b/iC3b (85–87). Interestingly, mice that are deficient in C3 or C3aR do not readily form NETs (87), emphasizing the link to complement. This is further evident in studies with COVID-19, where disease severity is tightly correlated with NETosis, reduced NET clearance, and augmented thrombin generation (88, 89). Release of TF positive NETs triggered by COVID-19 patient plasma is blocked by C5aR inhibitors (90) and pilot clinical studies with ant-C3 and anti-C5 agents suggest some protection against NETosis, inflammation and leukocytosis (91).

Since NETs comprise multiple constituents, including DNA, histones, various proteolytic enzymes, complement factors C3 and FB, lipids and other associated proteins (87, 92), they convey a myriad of properties that bridge coagulation and complement (**Figure 3**). The negatively charged NET components provide surfaces for activation of the contact pathway of coagulation, resulting in generation of FXIa, FXIIa and kallikrein (PKa) (93). NETs also release or expose functionally active TF and release TF positive extracellular vesicles that participate in the local amplification of thrombin generation. Histones H3 and H4 are major NET constituents. They act as DAMPs to activate complement, and to locally concentrate neutrophil elastase, cathepsin G, and myeloperoxidase (94), which themselves may activate complement. Histones also block the function of the serpin AT, promote autoactivation of prothrombin to thrombin (95), interfere with tPA-mediated plasmin generation (96), and bind to thrombomodulin and protein C to reduce thrombin-mediated generation of activated protein C (APC) (97). While APC normally digests histones, when NET-bound (98), the histones are resistant to degradation. It follows that NET-associated thrombi are more resistant to fibrinolysis (83, 99). Neutrophil-derived proteases and reactive oxygen species also exhibit pro-inflammatory and pro-coagulant properties, suppressing the functional expression of thrombomodulin (100), and inactivating natural inhibitors of the coagulation/complement systems, including TFPI (101). Overall, NETs underline the extensive interplay between coagulation and complement - truly major components in the seamless thromboinflammatory web.

### Contact activation and the kallikrein-kinin system

Triggering thrombin generation, complement activation and inflammation, the so-called intrinsic blood coagulation system and kallikrein-kinin systems, herein collectively referred to as the contact system (CS) (18). The CS represents a critical nexus within the thromboinflammatory web that is intimately connected to complement and coagulation. The major components of this system are high molecular weight kininogen (HK), plasma prekallikrein (PK) and FXII, which also assemble on negatively charged surfaces (e.g., aPL) in an auto-activating complex to produce FXIa, and thus downstream generation of thrombin (**Figure 1**). Based on clinical evidence that deficiency of FXII is not associated with excess bleeding, this pathway was not considered relevant in hemostasis-thrombosis. However, within the past couple of decades, the pathophysiologic relevance of this pathway in coagulation has been revealed in pre/clinical trials demonstrating that targeting FXII by various means, protects from thromboembolic disease (102–104). Its additional central role in inflammatory disorders is evident by the association of excess FXII activity with the sometimes life-threatening inflammatory disorder, hereditary angioedema (HAE) (102, 105). Indeed, anti-FXIIa treatments are holding promise as prophylaxis against angioedema due to C1-INH deficiency (106).

FXII normally circulates in the blood as a single polypeptide zymogen, that constitutively expresses a low level of activity (107), catalyzing its autoactivation, and activation of PK and FXII when in contact with a negatively charged surface, such as damaged blood vessels, invading pathogens, DNA, RNA (108), neutrophil extracellular traps (NETs) (109), anionic polysaccharides, polyphosphate, and activated endothelial cells and platelets (110, 111). FXIIa is then able to recruit HK bound to PK, leading to local cleavage of PK to generate the plasma serine protease, plasma kallikrein (PKa) (105). This in turn feeds back to generate more FXIIa and PKa. With sufficient FXIIa, FXI is activated, triggering thrombin generation and ultimately fibrin formation. Occurring at the initial site of vascular injury, FXIa is considered a supplementary source of thrombin generation. In that regard, the efficacy of FXII and FXI inhibitors, alone or in combination, to prevent thrombosis and inflammation are being explored by several groups (103, 112–114).

From within the HK/PKa/FXIIa complex, PKa proteolyses HK to form the pro-inflammatory, bradykinin (BK) (102), a nonapeptide that regulates vascular permeability and blood pressure (115). BK, tightly regulated by several peptidases (116) including, among others, carboxypeptidase B2 (CPB2) (117), also binds to G-protein coupled receptors on endothelial cells and activated leukocytes, stimulates nitric oxide and prostacyclin (PGI_2_) synthesis, and release of tPA from endothelial cells (118), which dampen coagulation, platelet activation and fibrin deposition. Exogenous tPA also increases PKa activity *via* a FXII-dependent manner, a notable observation that raises the consideration of inhibiting PKa during tPA-mediated thrombolysis in stroke to reduce brain hemorrhage and edema (119).

As noted previously, FXIIa also activates complement, cleaving C1r to trigger formation of the CP C3 convertase. Whether this pathway substantially contributes to complement activation in health and disease is unknown. More likely, particularly in C1-INH deficiency, heightened release of anaphylatoxins C3a and C5a could more readily be attributed to 1) excess PKa that can cleave C3 and FB, 2) lack of neutralization of C1r, C1s and MASPs, and 3) lack of inhibition of plasmin (116, 120).

Taken together, insights gained particularly from biochemical and genetic studies of the complex role of the CS in regulating coagulation, complement and inflammation, is uncovering exciting potential therapeutic targets, including for example, FXII/FXIIa, FXI/FXIa, BK, BK receptors, HK, PK, PKa, C1-INH, and gC1qR, for a wide range of thromboinflammatory disorders.

### Complement activation fragments, big and small

#### Terminal pathway complexes promote coagulation

Several observational studies have revealed that complement components, including C3, C4, C5a and FB are often found in thrombi (121) where they may initiate and sustain inflammation (122). C3 enhances clot stability and increases clot resistance to fibrinolysis by binding directly to fibrin, findings consistent with the prolonged bleeding time and delayed thrombosis post-injury in mice lacking C3 (123). Remarkably, few studies have further explored the molecular mechanisms by which these complement factors alter fibrin clot structure. There has been, however, considerable focus on the pore-forming C5b-9 MAC.

With MAC assembly, the membrane integrity of the unwanted cell or microorganism can be disrupted, thereby ensuring its destruction and elimination. Not surprisingly, altering the configuration and structure of membrane components, engages other biological systems, including coagulation. Platelets, endothelial cells and leukocytes are well-positioned to participate in complement activation, and indeed to be targeted as innocent bystanders, for destruction. Indeed, these cells are particularly sensitive to sublytic concentrations of C5b-9 (sC5b-9), which induces transbilayer flipping of aPL that can readily support coagulation activation through assembly of respective cofactor/enzyme complexes, and the ultimate generation of thrombin (124, 125). This occurs in concert with augmented secretion of VWF, P-selectin and pro-inflammatory cytokines, heightened expression of leukocyte adhesion molecules (126), and the release of microvesicles that are rich in C5b-9 and P-selectin, and complement inhibitors C1-INH, clusterin, CD55 and CD59 (127), as well as functionally active TF (128). C5b-9 has also recently been shown to directly trigger NETosis, that in turn induces neutrophil release of the pro-inflammatory cytokine, IL-17 (85). Most intriguing, these latter effects of C5b-9 were dampened by exosomes derived from mesenchymal stem cells in a CD59-dependent manner (85). The clinical relevance is being tested in pilot studies, as administration of mesenchymal stem cell exosomes that naturally accumulate abundant CD59 (an inhibitor of MAC formation), appears to dampen the inflammation associated with COVID-19 (129).

When combined with thrombin, the terminal pathway components result in enhanced activation and aggregation of platelets and the release of granules (130). This is similar to the apparent co-operativity of thrombin and C5 convertase in generating a more lytic MAC (C5b_T_-9) (131). In fact, even partial assembly to the point of C5b-7, by attaching to the outer leaflet of a target membrane, may be sufficient to trigger activation of TF on monocytic cells without inducing aPL exposure, but rather by promoting enzymatic decryption of TF *via* protein disulfide isomerase (132).

These prothrombotic, proinflammatory properties of the assembling complement terminal pathway components must be tightly regulated to prevent unwanted damage to healthy host cells. Indeed, there are several mechanisms: 1) Vitronectin binds to C5b-7, preventing it from binding to the outer membrane surface (133); 2) Clusterin interacts with C7, C8 and C9, diminishing the capacity of the C5b-9 complex to integrate into the membrane (134); 3) Polyphosphate binds to C6, destabilizing C5b,6, preventing C5b-8 and C5b-9 complexes from integrating into the membrane (135); and 4) On the cell surface, glycosylphosphatidylinositol (GPI)-linked CD59 binds to C8 and C9 and prevents C9 polymerization (136). Thus, MAC-triggered thrombo-inflammatory effects are coordinately dampened by shared regulatory pathways.

#### Anaphylatoxins delicately modulate coagulation

Activation of the complement system is accompanied by the liberation of potent anaphylatoxins, C3a and C5a (see **Table 2** for a summary of C5a biological effects). These relatively small peptides do more than just recruit inflammatory cells to sites of injury and infection. *Via* their widely expressed cognate receptors, C3aR for C3a, and C5aR1 and C5L2 for C5a, they exhibit multiple biological functions, including the promotion of coagulation and inflammation (137, 138).

**Table 2:** Activities of complement factor C5a that modulate thromboinflammation.

Cellular target	Cellular response
**Platelets**	triggers α-granule release of constituents (e.g., P-selectin, PF4, CD40L, PAF, integrin αIIbβ3, FV, FVIII, FXI, VWF, fibrinogen, HK, TFPI, PAI-1, Pg, MMPs, C1-INH, FH, CD55, CD59, CD46, clusterin, FD
	induces dense-granule release of constituents (e.g., serotonin, ADP, ATP, ionic calcium, polyphosphate)
	triggers lysosome release of constituents (e.g., hydrolases, cathepsins, elastases, glycosidases)
	induces exposure of P-selectin, a receptor for C3b and ligand for PSGL-1 to recruit neutrophils
	triggers exposure of gC1q-R, a receptor for C1q
	triggers exposure of CsA to which C1q, C4b binding protein and FH can bind
	triggers release of procoagulant microvesicles
**Endothelial cells**	upregulates leukocyte adhesion molecules
	increases secretion of P-selectin, VWF
	upregulates and activates cell surface TF
	suppresses expression of thrombomodulin
	damages the glycocalyx
	triggers release of procoagulant microvesicles
**Neutrophils**	chemoattractant
	cells are activated and induced to release proteolytic enzymes (e.g., elastase, cathepsins), ROS, chemoattractants and cytokines
	upregulates integrins to enhance migration and adhesion
	upregulates/activates TF
	augments expression of CR3 (CD11b) which facilitates adhesion, migration, phagocytosis
	triggers release of prothrombotic/proinflammatory NETs

Circulating quiescent platelets can become sensitized to stimulation by either C3a or C5a by minimal pre-activation, such as by adhesion to the subendothelium following vascular damage (139). By engaging their receptors, C3a and C5a induce platelet activation and aggregation (73, 140), triggering the release of C1q, C3, C4 and C5b-9 (141), surface exposure of chondroitin sulfate A, and α-granule release and surface exposure of P-selectin and the globular head receptor for C1q (gC1q-R). P-selectin is a receptor for C3b, and thus provides a means for assembly of the AP C3 convertases, while C1q binding to gC1q-R triggers the CP. Engagement of C1q with gC1q-R also induces conformational changes in the integrin GpIIbIIIa that supports platelet adhesion and aggregation (142) and promotes further P-selectin release with recruitment of leukocytes *via* interactions with P-selectin glycoprotein-1 (PSGL-1) (143). C3 that is hydrolyzed to C3(H2O) can also bind to the surface of activated platelets in the presence of leukocyte derived properdin, thereby facilitating formation of a platelet surface-localized AP C3(H_2_O)Bb convertase (63, 139). This platelet bound C3(H_2_O) may also serve as a ligand for leukocyte cell surface receptor CD11b/CD18, promoting platelet-leukocyte interactions and recruitment of activated TF-bearing, prothrombotic monocytes.

The LP has also been implicated in platelet-facilitated hemostatic mechanisms (144), as ficolins, MASP-1 and MASP-2 are found on the surface of activated platelets. This may be attenuated by the release from activated platelets of the thiol isomerase ERp57, which interferes with ficolin recognition *via* disruption of its multimerization (145). Indeed, there are several negative regulatory mechanisms that limit complement activation on the platelet surface, presumably to prevent the early demise of these important cells. Thus, the cell surface-expressed chondroitin sulfate A (CsA) can bind to C1q, C4b binding protein and FH, the latter two which dampen the immune response and limit further complement activation (146). Furthermore, platelet α-granules can release C1-INH, FH, CD55, CD59, CD46 and clusterin, while polyphosphate is released from dense granules. When secreted onto the surface of activated platelets, these can dampen MAC generation at different stages within the complement system. Although tightly regulated, the mechanisms that trigger P-selectin expression and accumulation of C3(H_2_O) on activated platelets enable C3a and C5a to sustain their own production and activate more platelets, leukocytes and endothelial cells to propagate coagulation and hemostasis.

With activation of complement *via* the CP/LP, C4a is liberated. This is believed to also have anaphylatoxic properties, but less potent than C3a and C5a. Nonetheless, C4a is interesting, as it interacts with platelets, binding to PAR1 and PAR4, thereby triggering intracellular events that promote their activation (147). A pathophysiologic contribution to hemostasis-thrombosis has not been established.

Endothelial cells also express receptors for C3a and C5a (74, 75, 148), engagement of which induces upregulation of leukocyte adhesion molecules, P-selectin, VWF and TF (149), suppression of thrombomodulin (150), and damage to the glycocalyx (151). Underlining a role for the engagement of C5aR in coagulation, in mice with a mutation in FH that causes diffuse microvascular and macrovascular thrombosis, blockade of C5aR protects against macrovascular thrombosis (152). Interestingly, lack of C3 or FD in those same FH mutant mice prevents all thrombosis (152). C5a also induces TF expression on neutrophils (153). Taken together, C3a and C5a and their receptors are pivotal, not only in recruiting circulating platelets and inflammatory leukocytes to the site of injury, but also in facilitating their participation in a thromboinflammatory response.

In addition to the likely roles of the anaphylatoxins, C3a, C5a and C4a in the function of primary hemostatic cells, platelets and endothelial cells, there is strong evidence that platelets, endothelial cells, neutrophils, and monocytes can be profoundly influenced by other “small” complement factors (154). This is exemplified by a recent report on the potential mechanisms underlying the thrombotic syndrome, heparin induced thrombocytopenia and thrombosis (HITT), a clinico-pathological arterial and venous thrombotic syndrome associated with the generation of heparin-dependent IgG anti-PF4 antibodies. These antibodies assemble into ultra-large immune complexes (ULICs) with heparin and PF4 on platelets. In a recently proposed model (155), the HITT ULICs bind to C1q and activate complement *via* the CP in the blood and on leukocytes, which leads to incorporation of C3c and C4d. These in turn engage complement receptors and Fcγ receptors on neutrophils and monocytes, upregulating TF expression and promoting coagulation and platelet activation/adhesion on endothelial cells, leading then to further thromboinflammation. Interventions that block C1 or C3 prevent leukocyte TF expression, while C5 blockade has no effect. Although yet to be confirmed *in vivo*, the model highlights the important role that each of the many proteolytic complement activation fragments may have in coagulation and the innate response to injury, and the potential therapeutic implications.

### Complementary enzymatic routes of complement and coagulation activation

#### Thrombin, more than a hemostatic factor

**Figure 4:**
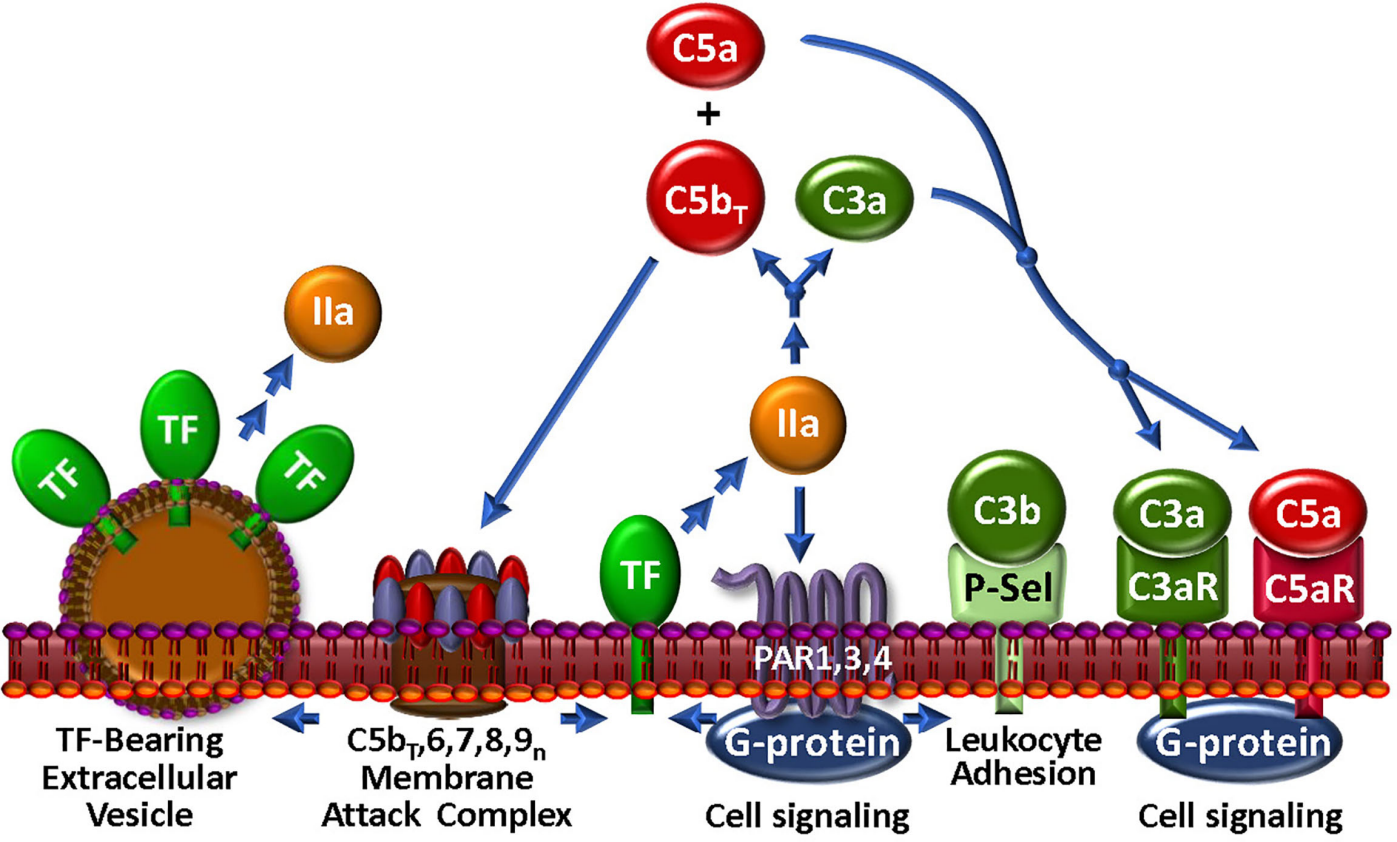
The Thrombin-Complement Web. Although the conditions under which this pathway exists *in vivo* remain to be shown, thrombin (IIa), in combination with the bona fide C5 convertase (not shown), cleaves C5 to C5b_T_ and C5a, which promote assembly of the MAC and an anaphylactic response, respectively. The MAC may induce the generation of extracellular vesicles, a well-documented source of TF. Thrombin also cleaves C3 to generate anaphylatoxin C3a. *Via* PARs 1, 3 and 4, thrombin triggers modulation of many cell types. In particular, P-selectin (P-Sel) can associate with C3b to upregulate the AP. Stimulation through PARs also facilitates expression of TF activity to further the thrombin-mediated effects on complement.

While thrombin is recognized as a central regulator of hemostasis (156), its broad substrate specificity within the humoral and cellular responses to vascular damage also interconnects thrombin to the host response to pathogen invasion through direct association with the complement network (**Figure 4**). Several laboratories have investigated the ability of thrombin to substitute as a C5 convertase, and claimed that thrombin, particularly at high concentrations, releases a fragment from C5 that is physically consistent with the generation of the anaphylatoxin, C5a (131, 157, 158). The C5 fragmentation milieu has biological activity in cell-based models (131, 157–159) and in some, but not all animal models (157, 160). A detailed biochemical investigation revealed that when the bona fide C5-convertase and thrombin are both present and active, as likely occurs with most injuries, non-canonical C5 and C5b cleavage products are generated that lead to the assembly of a more highly lytic C5b_T_-9 MAC (131), thereby augmenting the thromboinflammatory response to injury. This modified C5 was able to support binding of C6 and assembly of a MAC (161), potentially providing a “bypass” pathway that could be relevant in the strategic design of therapeutics. Contrary to its effects on C5, thrombin has limited capacity to generate a C3 convertase (162). However, thrombin may be able to enhance C3 convertase assembly indirectly *via* activation of pro-FD (68, 69), a fundamental accelerator of the AP.

Several of the preceding findings, mostly *in vitro* studies using various sources of C5, are not without controversy. *In vivo*, neither thrombin nor plasmin activated complement in a baboon sepsis model (160). More pointedly, elegant *in vitro* studies revealed that conformational changes in C5 in plasma from healthy donors, renders R947 inaccessible to cleavage by thrombin (163). It is not yet known whether protease cleavage sites, such as thrombin, within C5 (and C3) might be rendered accessible in diverse pathophysiological settings in which there are plasma disturbances in, for example, pH and electrolyte balance. In such situations, one or more of these non-canonical complement activation pathways may indeed be of clinical relevance, and thus potential therapeutic targets in disease. Further study is necessary!

Thrombin may also enhance complement *via* communication with cell surface receptors. Thus, exposure of platelets to thrombin causes deposition of C3 and the MAC (130, 164). This likely occurs *via* thrombin-triggered P-selectin release to the platelet surface, the latter which associates with C3b and enables assembly of the C3-convertase with subsequent C3 deposition and MAC formation (165). In line with the often opposing biological properties of thrombin, this also induces PAR1-mediated expression of the AP membrane control protein, CD55 (166) on vascular cells, indicating that spatial-temporal factors must be considered when evaluating the effects of these multi-versed proteases. Such observations also further underline a major theme of this review that the pathways involved in the thromboinflammatory response do not exist in isolation, but rather must finally be evaluated in the context of a more physiologic “web” of interactions.

Several other serine protease enzymes, particularly those involved in inflammation, have been reported to exhibit C5 convertase properties (167). The physiological relevance of most of these have not been established, but are worthy of consideration and further study. For example, neutrophil elastase directly cleaves C5, generating C5b,6 and a C5a-like moiety. Formation of the C5b-9 complex in this case, is limited by elastase-mediated hydrolysis of C6 (168). Cathepsin D that is released following severe tissue injury, has been directly correlated with C5 activation and generation of C5a (169). Factors IXa, Xa, XIa and PKa, have also been reported to cleave C5, bypassing the *bona fide* convertases in a C3-independent manner (157–159, 161). PKa can also cleave FH and FB (170, 171).

#### Plasmin: clot buster and complement activator

Several lines of evidence point to crosstalk between the fibrinolytic system and complement. For example, many chronic inflammatory disorders, such as atherosclerosis, exhibit colocalized and temporally overlapping activation of Pg with the accumulation of complement degradation products (172, 173). In addition to localization to the clot surface, plasmin generation may occur at sites of injury on endothelial cells, leukocytes or platelets - cells that are crucial for thrombin amplification and clot propagation - and where several receptors for Pg have been identified (174), including, for example, annexin A2-S100A10 (175) and Plg-Rkt (176). Upon activation either by tPA or urokinase plasminogen activator, cell-associated plasmin is likely involved in the recruitment and activation of inflammatory and immune cells and modulators (177). This is achieved *via* direct and indirect PAR-facilitated modulation of immune and inflammatory cells (177). However, it is also believed to occur in response to direct plasmin mediated proteolytic activation of C3 and C5, with release of the potent anaphylatoxins, C3a and C5a (158, 162, 178).

Interestingly, in spite of plasmin mediating the generation of C3a and C5a, there is little evidence for the subsequent assembly of their respective C3 or C5 convertases. This may be due to plasmin-mediated degradation of the key elements required for their formation (C3b, C5b, FB). There are, however, conflicting data as to whether plasmin activation of complement can provide an alternative route to generation and deposition of C5b-9 (162, 179). It is possible that such discordant observations are a function of differences in experimental setups, as well as the spatial-temporal availability of inhibitors and negative regulators of plasmin and complement (158).

The action of plasmin and plasmin-like proteases on the complement system likely extend further. As an evolutionary adaptation, plasmin-like activity is facilitated by bacteria-encoded activators (e.g. staphylokinase) directly on a pathogen surface. In this locale, previously deposited opsonins C3b and iC3b can be removed from the pathogen surface by the acquired proteolytic activity (180). Indeed, plasmin can cleave iC3b, yielding C3d/g-like peptides that bind to complement receptors CR3/4 and CR2 on leukocytes, thereby dampening phagocytosis and enhancing macrophage secretion of IL-12, respectively (174, 181, 182). The complement-fixing fragment of IgG (i.e., Fc) is furthermore stripped from the pathogen by these plasmin-like proteases. Overall, opsonization and further deposition of C3b by the classical and alternative pathways of complement are thus prevented. These immune-evading mechanisms highlight the complex and intertwined roles of plasmin and complement in pathogen surveillance.

#### MASPs: Complement activator or coagulation activator?

**Figure 5:**
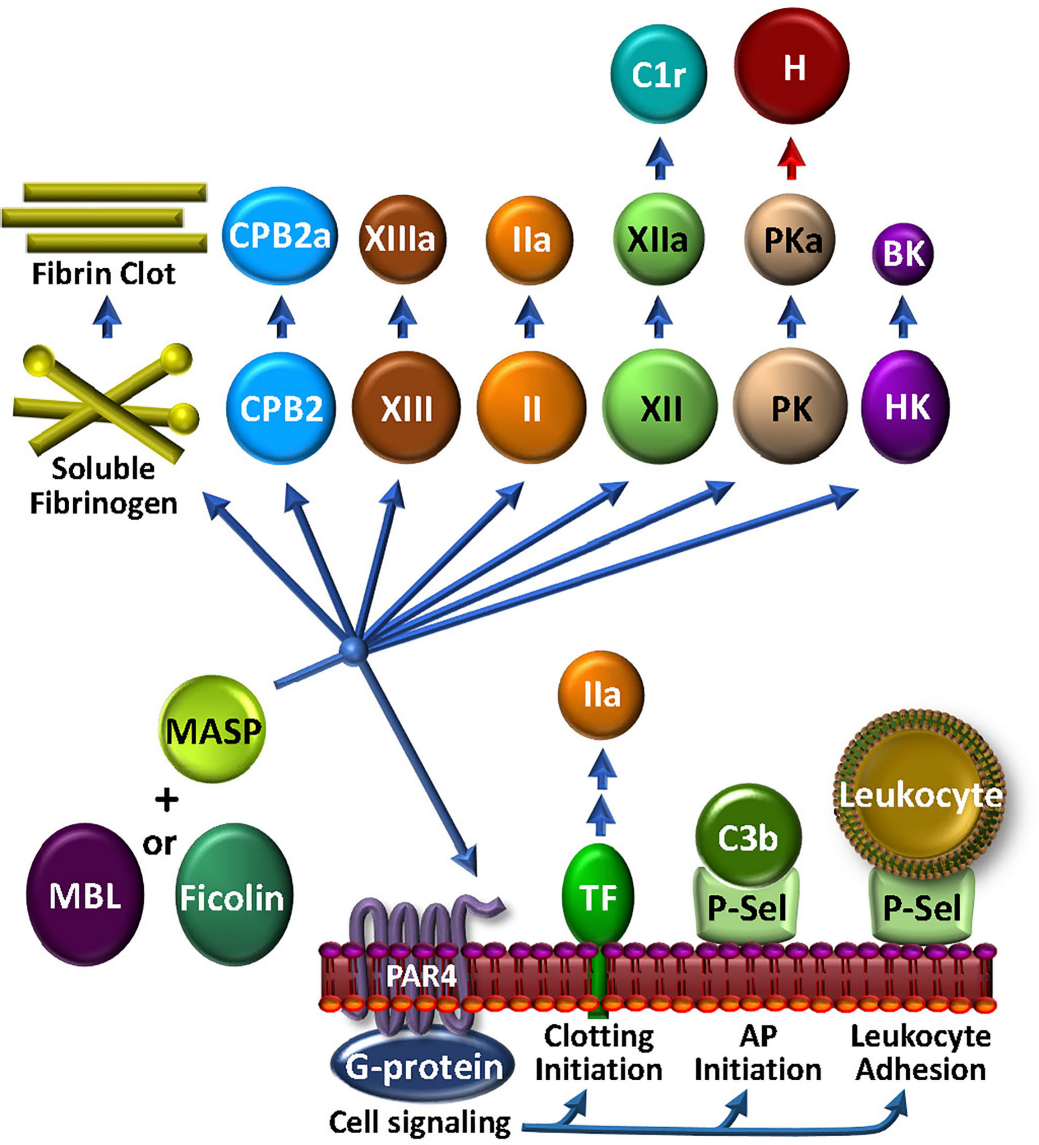
The MASP-Coagulation Web: MASPs may be either solution phase or surface bound, possibly associated with MBL, ficolin or collectin. Once triggered by engagement with an appropriate foreign lectin or other ligand, the activated MASP may stimulate cells *via* PAR4, leading to the availability of procoagulant TF or cell adhesive P-selectin activity. The latter may also participate to enhance AP C3 convertase assembly and function. MASPs have a wide array of circulating coagulation factor substrates, which may lead to bradykinin (BK) and kallikrein (PKa) that can inactivate factor H (H) and consequently up-regulate the AP, FXIIa that can activate C1r, thrombin (IIa), FXIIIa, carboxypeptidase 2 (CPB2), and fibrin production.

The most abundant of the MBL-associated serine proteases in complement, MASP1, is required for activation of the LP. However, MASP1 has a promiscuous catalytic site that is more like thrombin than its CP C2-activating counterparts, C1r and C1s (183). MASP1 directly activates endothelial cells *via* PAR4 (184), thereby triggering intracellular signaling cascades that promote a pro-inflammatory response (59, 185) (**Figure 5**). In purified *in vitro* systems, MASP1 also activates prothrombin (186) and CPB2, cleaves fibrinogen to fibrin monomers, activates FXIII, and generates BK from HK. MASP2 is more restricted, but similarly cleaves prothrombin to thrombin (187) and activates FXII and PK (188). Once activated to FXIIa by either MASP-mediated mechanisms or the canonical contact phase mechanism, FXIIa can cleave C1r, triggering feedback amplification of complement *via* the CP (189). With the most limited substrate range of the MASPs, MASP-3 cleaves pro-factor D into factor D, thereby triggering the AP, and thus not wandering beyond complement (69).

The physiologic relevance of these apparent MASP-mediated proteolytic activities in coagulation and complement have not been confirmed. However, while MASP1 and MASP2 are >100-fold less active than the corresponding bona fide coagulation enzymes (FXa and thrombin) in cleaving prothrombin and fibrinogen, there are strong data supporting their contribution to thrombosis in various clinical situations. Mice deficient in either MASP1 or MBL are resistant to thrombosis in a carotid artery injury model (190). This is in line with clinical studies, in which patients with low MBL plasma levels had a lower risk of deep venous thrombosis (191). In light of the LP being implicated in contributing to the thromboinflammation associated with COVID-19 (183), major efforts are underway to identify agents that can block MASP2 (192). Phase 3 studies are ongoing to test the efficacy of a MASP2 inhibitor in protecting against the thrombotic microangiopathy associated with hematopoietic stem cell transplants (193). Overall, the story of MASPs in coagulation and complement and thromboinflammation is still unfolding.

### Braking systems that affect complement and coagulation

As innate responders to bleeding, foreign or damaged cells, cellular by-products or invading pathogens, if unchecked, coagulation and complement activation may cause unwanted bystander damage to the host, leading to a proinflammatory and prothrombotic state with organ dysfunction and failure. Thus, tight regulation of these systems in a coordinated manner is essential. There are, indeed, multiple regulatory mechanisms at several steps - many of which are shared or overlap - these being achieved *via* membrane anchored and fluid-phase regulators.

#### Regulation of complement and coagulation proteases by serpins and non-serpins

Once activated, the proteases of complement, coagulation, fibrinolysis and the CS are under constant surveillance by multiple inhibitory mechanisms. Intrinsic to plasma are members of the serine protease inhibitor (serpin) homology family. Serpins deceive a protease by presenting a central reactive loop that resembles the target substrate. Upon cleavage, a gross conformational change traps the protease in an irreversible complex and is subsequently cleared (194). Serpins typically neutralize more than one type of protease. Perhaps the most important of these in coagulation is antithrombin (AT), which is the primary inhibitor of thrombin, FXa and FIXa (195, 196). Functional deficiencies in AT substantially increase the risk of thrombosis (197). Notably, AT also inhibits MASP1 and MASP2 in the LP (198), although its pathophysiologic importance in complement activation is not known.

Clearly demonstrating a complement-coagulation overlap, C1 inhibitor (C1-INH) is a broad-specificity serpin with special significance for regulating both the contact phase branch of coagulation (199) and the CP of complement. C1-INH regulates the CS by controlling PK activation, and neutralizing the activities of PKa and FXIIa, likely better for the former than the latter (200). It also inhibits plasmin (12). In so doing, C1-INH reduces generation of the potent pro-inflammatory BK (102, 115), and within the hemostatic network, contributes to downstream inactivation of FXIa (201) and plasmin (202), thus affecting functional protease regulation in several pathways, and cell signaling *via* the PARs.

Within complement, C1-INH blocks several proteases, including C1r and C1s of the CP (203), and MASP1 and MASP2 of the LP (204), dampening generation of the CP/LP C3 convertases. In the complement system, and the coagulation system, C1-INH function is potentiated by polyanions, including heparan sulfate and polyphosphate (205–207), the latter which is a major procoagulant at several steps, including a key trigger for the CS (208–210). Independent of its function as a serpin, C1-INH also directly interacts with C3b, preventing binding of FB, and thus the formation of the AP C3 convertase (211). Based on the genetic association of C1-INH deficiency with hereditary angioedema, C1-INH should most prominently be viewed as a strategic piece for controlling BK formation (212). Notably however, its physiologic contribution to regulating coagulation has recently been revealed, with evidence - contrary to previous claims - that C1-INH deficiency is in fact, associated with an increased risk of thrombosis (213).

Non-serpin protease inhibitors are also important for the regulation of coagulation and complement. The only endogenous inhibitor of the extrinsic tenase is tissue factor pathway inhibitor (TFPI) (34, 214, 215), which may be GPI-linked and therefore a proportion is cell surface-associated. TFPI forms a high affinity quaternary complex with TF/FVIIa/FXa. Microvascular endothelial cells, monocytes, platelets and smooth muscle cells constitutively express TFPI (216) and the non-GPI-modified isoform is identified in plasma. In addition to its role in coagulation, TFPI inhibits the LP by interfering with MASP2 cleavage of C4 and C2 (217). Interestingly, the acquired clonal hematologic disorder PNH, that is associated with excess complement activation and thrombosis, is caused by a mutation in the phosphatidylinositol glycan class A gene that encodes the protein necessary for GPI anchoring of several proteins, including in particular the complement negative regulators CD55 andCD59, but also TFPI. Thus, TFPI would predictably be absent in affected hematopoietic cells in PNH, a situation that might partially explain the heightened risk of thrombosis in these patients.

MAP-1 (aka Map44) and sMAP are alternative, non-enzymatic splice forms of MASP1/3 and MASP2, respectively. These compete for MASP binding to MBL, ficolins and collectins, thereby interfering with formation of the LP PRM (218). The relevance of these interactions, not only in complement activation and regulation, but also in coagulation, remain unclear.

Sushi domain-containing protein 4 (SUSD4) is a complement control protein that, due to alternative splicing, may be expressed as an integral membrane protein or a soluble protein. Although the mechanisms and physiologic relevance are incompletely understood, both forms inhibit formation of the C3 convertase, the soluble form targeting the CP and LP, and the membrane form, targeting the CP and AP (219). A potential direct role for SUS4 in coagulation has not been studied.

#### Convertases, FH and the AP

Although the convertases themselves are self-regulated by inherent instability, with half-lives in the range of minutes (220, 221), additional factors are essential to facilitate disruption of the integrity of the convertases, thereby preventing bystander injury to healthy host cells. Factor H (FH) is arguably the most potent and versatile AP negative regulator (222, 223) that targets C3 convertase, utilizing 3 distinct mechanisms to dampen complement activation. FH binds both to C3b and to host cells *via* defined molecular structures (224), whereupon it, 1) competes with FB binding to C3b, thereby preventing convertase assembly; 2) enhances serine protease factor I (FI)-mediated proteolysis of surface-bound C3b and C4b to iC3b/C3dg and iC4b/C4d, respectively, rendering them incapable of assembling a functional C3 convertase; and 3) accelerates decay of the C3b/Bb convertase. This latter decay accelerating mechanism is also used by CR1, CD55, FH-like protein-1 and the transmembrane glycoprotein receptor CD46. All of these, except CD55, also promote specific FI-mediated proteolysis of C3b and C4b (225). The liberated fragments, while incapable of assembling into a convertase, act as opsonins and trigger phagocytosis and adaptive immune responses. Interestingly, in the setting of inflammation or sepsis, C3b may be modified by a platelet-released kinase (226) such that it is resistant to the properties of FH (227). Whether this affects the function of the other cofactors for FI, is not known.

Recent *in vitro* studies have uncovered a potential co-regulatory complement-hemostasis relationship between FH and FXIa (228). FXIa cleaves FH at a site involved in enhancing the risk of age-related macular degeneration (229), reducing FH binding to endothelial cells, its cofactor activity in FI-mediated inactivation of C3b, and its C3b/Bb decay function. The FXa proteolytic activity was increased in the presence of polyphosphate, a prothrombotic polyanion that also binds to FH and exhibits anti-complement activity (135, 206). FH inhibits FXI activation by thrombin or FXIIa. Interestingly, FH has recently been found in plasma, complexed with FXIIa (230). Whether these interconnected regulatory mechanisms are relevant in health and thromboinflammatory disease is not yet known.

That FH and its protein binding-partners in the AP provide a bridge between coagulation and complement, is evident from extensive studies of patients with the thrombotic microangiopathy, atypical hemolytic uremic syndrome (aHUS). Patients with functional mutations in FH are at increased risk of developing aHUS (231–233); and indeed, loss-of-function or gain-of-function mutations in AP components that result in excess complement activation account for 60-70% of all cases of aHUS (234, 235). Interestingly, consistent with a potential role of Pg as a cofactor for FI-mediated inactivation of C3b (179), Pg-deficient variants have also been linked to aHUS (236). Most notably, and indicative of the role of complement hyperactivation triggering the thrombosis, is the protection afforded to almost all aHUS patients, with the anti-C5 antibody, eculizumab (13).

#### FH and thrombomodulin: Cooperative regulation of coagulation and complement

Thrombomodulin (TM) is a multidomain, transmembrane glycoprotein expressed on the surface of all vascular endothelial cells. It is a critical cofactor for thrombin-mediated activation of protein C (PC) to generate APC, catalyzing the reaction by ~1000-fold (237). Deficiency of PC augments the risk of deep vein thrombosis and thromboembolism (238) and the response to inflammatory stimuli. In a similar manner as for PC, TM also augments thrombin-mediated generation of the antifibrinolytic CPB2 (239), which also functions to inactivate pro-inflammatory mediators, BK, osteopontin, and the critical anaphylatoxins, C3a and C5a (117). The anaphylatoxins may also be proteolytically inactivated by MMP-12, activation of which is enhanced by CPB2-mediated prolongation of plasmin generation, which further produces complement opsonic fragments (240). TM reaches even further into the complement network by enhancing FI-mediated inactivation of C3b in the presence of FH (241–243). Overall, by sequestering thrombin from its myriad prothrombotic, pro-inflammatory and complement-activating effects, and by inactivating C3a, C5a and BK, and by augmenting the properties of FH, TM provides a critical and clinically relevant bridge between coagulation and complement that additionally integrates into the thromboinflammatory web *via* its diverse properties in cell proliferation, leukocyte trafficking, and endothelial function [reviewed (244)].

#### FH and VWF interconnect complement and coagulation

VWF plays key roles in hemostasis and complement activation. Synthesized by megakaryocytes and endothelial cells in an ultra large multimeric form (ULVWF), it is secreted by endothelial cells following stress or injury, whereupon it is normally cleaved into smaller units by the enzyme ADAMTS13. However, with insufficient cleavage, the ULVWF multimers accumulate and promote excess platelet adhesion and aggregation and the formation of microvascular thrombi. This is manifest as the thrombotic microangiopathy, thrombotic thrombocytopenia purpura (245). ULVWF multimers provide a binding site for C3b and thus assembly of the AP C3 convertase (246), with potential subsequent activation of complement and bystander injury to neighboring cells. FH has been shown to colocalize with VWF in endothelial cell Weibel-Palade bodies, strongly suggesting a functional relationship. Although controversial, evidence indicates that FH may facilitate ADAMTS13-mediated proteolysis of the ULVWF into monomers and dimers (247–250), in addition to its established role as an AP regulator. Not only are these smaller VWF forms less amenable to C3b binding, but they may also act as a cofactor for C3b inactivation by FI (251). Thus, VWF is reciprocally indicated as a negative regulator of complement.

## Concluding remarks

The preceding review highlights some of the complex interactions between complement and coagulation and how these interface with the apparently seamless web that comprise other critical pathways that are involved in the thromboinflammatory response to injury and infection. The value in deciphering the intricacies of this web of molecular and cellular relationships on disease outcome, is underlined by the overwhelming success of the terminal pathway anti-C5 antibodies, eculizumab and ravulizumab, in preventing the devastating thrombotic manifestations of aHUS and PNH (13, 14, 193). It is also evident by exciting new advances in our understanding of these interactive pathways, as several newer agents that target, for example, MASP2, FB, FD, C3, the C5a-C5aR axis, BK, BR, FXII/FXIIa, FXI/FXIa, polyanions, platelets, neutrophils and NETs, are being evaluated in clinical studies that are at various stages of development, and in many cases with promising results (reviewed in (193, 252, 253). And more are on the horizon. Varying responses indicate that tailored and personalized interventional strategies will undoubtedly be required to optimally prevent unwanted thromboinflammatory responses where coagulation and complement participate. This will necessarily entail continued research efforts to tease apart the intricacies of the web - examining pathways in isolation and in more complex environments - to uncover novel techniques and therapies, diagnostic tools and biomarkers for a wide range of disorders that reside within and extend beyond those traditionally viewed as coagulation or complement.

## Author contributions

EP, AL and EC researched and wrote the manuscript. All authors contributed to the article and approved the submitted version.

## Funding

EC is supported by the Canadian Institutes of Health Research (CIHR), the Natural Sciences and Engineering Research Council of Canada (NSERC), the Canada Foundations for Innovation (CFI), CanVECTOR and the Canada Research Chairs Program. He is an Adjunct Scientist with the Canadian Blood Services. EP is supported by the Canadian Blood Services and is supported by the Heart and Stroke Foundation of Canada and the CIHR. AL is supported by a MITACs Accelerate Postdoctoral Fellowship IT26813.

## Conflict of interest

The authors declare that the research was conducted in the absence of any commercial or financial relationships that could be construed as a potential conflict of interest.

## Publisher’s note

All claims expressed in this article are solely those of the authors and do not necessarily represent those of their affiliated organizations, or those of the publisher, the editors and the reviewers. Any product that may be evaluated in this article, or claim that may be made by its manufacturer, is not guaranteed or endorsed by the publisher.

## References

[1] Owen C. A history of blood coagulation. Rochester, Minnesota: Mayo Foundation (2001).

[2] Beck EA. The chemistry of blood coagulation: a summary by Paul morawitz (1905). Thromb haemostasis (1977) 37:376–9. doi: 10.1055/s-0038-1649245 578019

[3] Graham-Smith GS. George Henry Falkiner Nuttall: (5 July 1862-16 December 1937). J Hyg (Lond) 38(1938):129–i4. doi: 10.1017/S0022172400010986 PMC2199554 20475415

[4] Buchner H. [Brief overview of the development of bacteriology since naegeli's involvement in it]. Münchener Medizinische Wochenschriftlrm; (1891) 38:435–7.

[5] Bardhan M Kaushik R. Physiology, complement cascade. Treasure Island (FL: StatPearls (2022). 31855355

[6] Wising P. The identity of prothrombin as the midpiece of complement. Acta Med Scand (1938) 94:506–9. doi: 10.1111/j.0954-6820.1938.tb09508.x

[7] Ratnoff OD Lepow IH. Some properties of an esterase derived from preparations of the first component of complement. J Exp Med (1957) 106:327–43. doi: 10.1084/jem.106.2.327 PMC2136749 13449241

[8] Wedgwood RJ Pillemer L. The nature and interactions of the properdin system. Acta Haematol (1958) 20:253–9. doi: 10.1159/000205491 13582571

[9] Cooper NR. Complement: a nostalgic journey the Hans j. Muller-eberhard memorial lecture, Honolulu, June 14, 2004. Mol Immunol (2006) 43:487–95. doi: 10.1016/j.molimm.2005.04.018 15961158

[10] Davie EW Ratnoff OD. Waterfall sequence for intrinsic blood clotting. Science (1964) 145:1310–2. doi: 10.1126/science.145.3638.1310 14173416

[11] Pillemer L Ratnoff OD Blum L Lepow IH. The inactivation of complement and its components by plasmin. J Exp Med (1953) 97:573–89. doi: 10.1084/jem.97.4.573 PMC2136289 13052820

[12] Ratnoff OD. Some relationships among hemostasis, fibrinolytic phenomena, immunity, and the inflammatory response. Adv Immunol (1969) 10:145–227. doi: 10.1016/S0065-2776(08)60417-4 4242699

[13] Gruppo RA Rother RP. Eculizumab for congenital atypical hemolytic-uremic syndrome. New Engl J Med (2009) 360:544–6. doi: 10.1056/NEJMc0809959 19179329

[14] Hillmen P Young NS Schubert J Brodsky RA Socie G Muus P. The complement inhibitor eculizumab in paroxysmal nocturnal hemoglobinuria. New Engl J Med (2006) 355:1233–43. doi: 10.1056/NEJMoa061648 16990386

[15] W.H. Organization. Global atlas on cardiovascular disease prevention and control. Geneva: WHO (2011).

[16] Pryzdial ELG Lee FMH Lin BH Carter RLR Tegegn TZ Belletrutti MJ. Blood coagulation dissected. Transfus. Apher. Sci (2018) 57:449–57. doi: 10.1016/j.transci.2018.07.003 30049564

[17] Versteeg HH Heemskerk JW Levi M Reitsma PH. New fundamentals in hemostasis. Physiol Rev (2013) 93:327–58. doi: 10.1152/physrev.00016.2011 23303912

[18] Schmaier AH. The contact activation and Kallikrein/Kinin systems: Pathophysiologic and physiologic activities. J Thromb Haemost (2016) 14:28–39. doi: 10.1111/jth.13194 26565070

[19] Protty MB Jenkins PV Collins PW O'Donnell VB. The role of procoagulant phospholipids on the surface of circulating blood cells in thrombosis and haemostasis. Open Biol (2022) 12:210318. doi: 10.1098/rsob.210318 35440201 PMC9019515

[20] Macfarlane RG. An enzyme cascade in the blood clotting mechanism, and its function as a biochemical amplifier. Nature (1964) 202:498–9. doi: 10.1038/202498a0 14167839

[21] d'Alessandro E Becker C Bergmeier W Bode C Bourne JH Brown H. Thrombo-inflammation in cardiovascular disease: An expert consensus document from the third maastricht consensus conference on thrombosis. Thromb haemostasis (2020) 120:538–64. doi: 10.1055/s-0040-1708035 32289858

[22] Rosenberg RD Rosenberg JS. Natural anticoagulant mechanisms. J Clin Invest (1984) 74:1–6. doi: 10.1172/JCI111389 6330171 PMC425178

[23] Clemetson KJ. Platelets and primary haemostasis. Thromb Res (2012) 129:220–4. doi: 10.1016/j.thromres.2011.11.036 22178577

[24] Lenoir G D'Ambrosio JM Dieudonne T Copic A. Transport pathways that contribute to the cellular distribution of phosphatidylserine. Front Cell Dev Biol (2021) 9:737907. doi: 10.3389/fcell.2021.737907 34540851 PMC8440936

[25] Heijnen H van der Sluijs P. Platelet secretory behaviour: as diverse as the granules ... or not? J Thromb Haemost (2015) 13:2141–51. doi: 10.1111/jth.13147 26391322

[26] Flaumenhaft R Sharda A. Platelet secretion. In: Michelson AE Cattaneo M Frelinger AL Newman PJ, editors. Platelets. (Amsterdam:Elsevier) (2019). p. 349–70.

[27] Bach RR. Tissue factor encryption. Arteriosclerosis thrombosis Vasc Biol (2006) 26:456–61. doi: 10.1161/01.ATV.0000202656.53964.04 16397140

[28] Ansari SA Pendurthi UR Rao LVM. Role of cell surface lipids and thiol-disulphide exchange pathways in regulating the encryption and decryption of tissue factor. Thromb haemostasis (2019) 119:860–70. doi: 10.1055/s-0039-1681102 PMC7805104 30861549

[29] Rao LV Rapaport SI. Activation of factor VII bound to tissue factor: a key early step in the tissue factor pathway of blood coagulation. Proc Natl Acad Sci U. S. A (1988) 85:6687–91. 10.1073/pnas.85.18.6687 PMC282042 3261869

[30] Krishnaswamy S. The interaction of human factor VIIa with tissue factor. J Biol Chem (1992) 267:23696–706. doi: 10.1016/S0021-9258(18)35894-0 1429710

[31] Pryzdial ELG. Maestro tissue factor reaches new hEIGHT. Blood (2017) 130:1604–5. doi: 10.1182/blood-2017-08-798520 28983017

[32] Krishnaswamy S Field KA Morrissey JH Edgington TS Mann KG. Activation of factor X by the extrinsic pathway. Thromb Haemostasis (1991) 65:296.2048053

[33] Kamikubo Y Mendolicchio GL Zampolli A Marchese P Rothmeier AS Orje JN. Selective factor VIII activation by the tissue factor-factor VIIa-factor xa complex. Blood (2017) 130:1661–70. doi: 10.1182/blood-2017-02-767079 PMC5630012 28729433

[34] Mast AE Ruf W. Regulation of coagulation by tissue factor pathway inhibitor: Implications for hemophilia therapy. J Thromb Haemost (2022) 20:1290–300. doi: 10.1111/jth.15697 PMC9314982 35279938

[35] Olson ST Richard B Izaguirre G Schedin-Weiss S Gettins PG. Molecular mechanisms of antithrombin-heparin regulation of blood clotting proteinases. a paradigm for understanding proteinase regulation by serpin family protein proteinase inhibitors. Biochimie (2010) 92:1587–96. doi: 10.1016/j.biochi.2010.05.011 PMC2974786 20685328

[36] Schuijt TJ Bakhtiari K Daffre S Deponte K Wielders SJ Marquart JA. Factor xa activation of factor V is of paramount importance in initiating the coagulation system: lessons from a tick salivary protein. Circulation (2013) 128:254–66. doi: 10.1161/CIRCULATIONAHA.113.003191 PMC3826089 23817575

[37] Krishnaswamy S Nesheim ME Pryzdial ELG Mann KG. Assembly of the prothrombinase complex. Methods enzymol (1994) 222:260–80. doi: 10.1016/0076-6879(93)22018-B 8412798

[38] Hoffman M Monroe 3DM. A cell-based model of hemostasis. Thromb haemostasis (2001) 85:958–65. doi: 10.1055/s-0037-1615947 11434702

[39] Macfarlane SR Seatter MJ Kanke T Hunter GD Plevin R. Proteinase-activated receptors. Pharmacol Rev (2001) 53:245–82. 11356985

[40] Vu T-KH Hung DT Wheaton VI Coughlin SR. Molecular cloning of a functional thrombin receptor reveals a novel proteolytic mechanism of receptor activation. Cell (1991) 64:1057–68. doi: 10.1016/0092-8674(91)90261-V 1672265

[41] Coughlin SR. Protease-activated receptors in hemostasis, thrombosis and vascular biology. J Thromb Haemostasis (2005) 3:1800–14. doi: 10.1111/j.1538-7836.2005.01377.x 16102047

[42] Zelaya H Rothmeier A Ruf W. Tissue factor at the crossroad of coagulation and cell signaling. J Thromb Haemost (2018) 26:1941–52. doi: 10.1111/jth.14246 30030891

[43] Keragala CB Medcalf RL. Plasminogen: an enigmatic zymogen. Blood (2021) 137:2881–9. doi: 10.1182/blood.2020008951 33735914

[44] Kuliopulos A Covic L Seeley SK Sheridan PJ Helin J Costello CE. Plasmin desensitization of the PAR1 thrombin receptor: kinetics, sites of truncation, and implications for thrombolytic therapy. Biochemistry (1999) 38:4572–85. doi: 10.1021/bi9824792 10194379

[45] Shimazu H Munakata S Tashiro Y Salama Y Dhahri D Eiamboonsert S. Pharmacological targeting of plasmin prevents lethality in a murine model of macrophage activation syndrome. Blood (2017) 130:59–72. doi: 10.1182/blood-2016-09-738096 28325863

[46] Syrovets T Jendrach M Rohwedder A Schule A Simmet T. Plasmin-induced expression of cytokines and tissue factor in human monocytes involves AP-1 and IKKbeta-mediated NF-kappaB activation. Blood (2001) 97:3941–50. doi: 10.1182/blood.V97.12.3941 11389038

[47] Domotor E Bartha K Machovich R Adam-Vizi V. Protease-activated receptor-2 (PAR-2) in brain microvascular endothelium and its regulation by plasmin and elastase. J neurochem (2002) 80:746–54. doi: 10.1046/j.0022-3042.2002.00759.x 11948237

[48] Werb Z. ECM and cell surface proteolysis: regulating cellular ecology. Cell (1997) 91:439–42. doi: 10.1016/S0092-8674(00)80429-8 9390552

[49] Carmeliet P Moons L Lijnen R Baes M Lemaitre V Tipping P. Urokinase-generated plasmin activates matrix metalloproteinases during aneurysm formation. Nat Genet (1997) 17:439–44. doi: 10.1038/ng1297-439 9398846

[50] Heissig B Lund LR Akiyama H Ohki M Morita Y Romer J. The plasminogen fibrinolytic pathway is required for hematopoietic regeneration. Cell Stem Cell (2007) 1:658–70. doi: 10.1016/j.stem.2007.10.012 PMC2646407 18371407

[51] Heuberger DM Schuepbach RA. Protease-activated receptors (PARs): mechanisms of action and potential therapeutic modulators in PAR-driven inflammatory diseases. Thromb J (2019) 17:4. doi: 10.1186/s12959-019-0194-8 30976204 PMC6440139

[52] Ricklin D Lambris JD. Complement-targeted therapeutics. Nat Biotechnol (2007) 25:1265–75. doi: 10.1038/nbt1342 PMC2966895 17989689

[53] Pouw RB Ricklin D. Tipping the balance: intricate roles of the complement system in disease and therapy. Semin immunopathol (2021) 43:757–71. doi: 10.1007/s00281-021-00892-7 PMC8547127 34698894

[54] Conway EM. Complement-coagulation connections. Blood Coagul Fibrinolysis (2018) 29:243–51. doi: 10.1097/MBC.0000000000000720 29517503

[55] Roh JS Sohn DH. Damage-associated molecular patterns in inflammatory diseases. Immune Netw (2018) 18:e27. doi: 10.4110/in.2018.18.e27 30181915 PMC6117512

[56] Kidmose RT Laursen NS Dobo J Kjaer TR Sirotkina S Yatime L. Structural basis for activation of the complement system by component C4 cleavage. Proc Natl Acad Sci United States America (2012) 109:15425–30. doi: 10.1073/pnas.1208031109 PMC3458355 22949645

[57] Wijeyewickrema LC Yongqing T Tran TP Thompson PE Viljoen JE Coetzer TH. Molecular determinants of the substrate specificity of the complement-initiating protease, C1r. J Biol Chem (2013) 288:15571–80. doi: 10.1074/jbc.M113.451757 PMC3668718 23589288

[58] Degn SE Jensen L Hansen AG Duman D Tekin M Jensenius JC. Mannan-binding lectin-associated serine protease (MASP)-1 is crucial for lectin pathway activation in human serum, whereas neither MASP-1 nor MASP-3 is required for alternative pathway function. J Immunol (2012) 189:3957–69. doi: 10.4049/jimmunol.1201736 22966085

[59] Dobo J Schroeder V Jenny L Cervenak L Zavodszky P Gal P. Multiple roles of complement MASP-1 at the interface of innate immune response and coagulation. Mol Immunol (2014) 61:69–78. doi: 10.1016/j.molimm.2014.05.013 24935208

[60] Lachmann PJ Hughes-Jones NC. Initiation of complement activation. Springer Semin immunopathol (1984) 7:143–62. doi: 10.1007/BF01893018 6495149

[61] Nilsson B Ekdahl KN. The tick-over theory revisited: is C3 a contact-activated protein? Immunobiology (2012) 217:1106–10. doi: 10.1016/j.imbio.2012.07.008 22964236

[62] Bexborn F Andersson PO Chen H Nilsson B Ekdahl KN. The tick-over theory revisited: formation and regulation of the soluble alternative complement C3 convertase (C3(H2O)Bb). Mol Immunol (2008) 45:2370–9. doi: 10.1016/j.molimm.2007.11.003 PMC2701500 18096230

[63] Saggu G Cortes C Emch HN Ramirez G Worth RG Ferreira VP. Identification of a novel mode of complement activation on stimulated platelets mediated by properdin and C3(H2O). J Immunol (2013) 190:6457–67. doi: 10.4049/jimmunol.1300610 PMC3784323 23677468

[64] Des Prez RM Bryan CS Hawiger J Colley DG. Function of the classical and alternate pathways of human complement in serum treated with ethylene glycol tetraacetic acid and MgCl2-ethylene glycol tetraacetic acid. Infection Immun (1975) 11:1235–43. doi: 10.1128/iai.11.6.1235-1243.1975 PMC415205 806523

[65] White RT Damm D Hancock N Rosen BS Lowell BB Usher P. Human adipsin is identical to complement factor d and is expressed at high levels in adipose tissue. J Biol Chem (1992) 267:9210–3. doi: 10.1016/S0021-9258(19)50409-4 1374388

[66] Beatty DW Davis AE Cole 3FS Einstein LP Colten HR. Biosynthesis of complement by human monocytes. Clin Immunol Immunopathol (1981) 18:334–43. doi: 10.1016/0090-1229(81)90126-4 6910430

[67] Takahashi M Ishida Y Iwaki D Kanno K Suzuki T Endo Y. Essential role of mannose-binding lectin-associated serine protease-1 in activation of the complement factor d. J Exp Med (2010) 207:29–37. doi: 10.1084/jem.20090633 20038603 PMC2812541

[68] Oroszlan G Kortvely E Szakacs D Kocsis A Dammeier S Zeck A. MASP-1 and MASP-2 do not activate pro-factor d in resting human blood, whereas MASP-3 is a potential activator: Kinetic analysis involving specific MASP-1 and MASP-2 inhibitors. J Immunol (2016) 196:857–65. doi: 10.4049/jimmunol.1501717 26673137

[69] Dobo J Szakacs D Oroszlan G Kortvely E Kiss B Boros E. MASP-3 is the exclusive pro-factor d activator in resting blood: the lectin and the alternative complement pathways are fundamentally linked. Sci Rep (2016) 6:31877. doi: 10.1038/srep31877 27535802 PMC4989169

[70] Fearon DT Austen KF. Properdin: binding to C3b and stabilization of the C3b dependent C3 convertase. J Exp Med (1975) 142:856–63. doi: 10.1084/jem.142.4.856 PMC2189935 1185108

[71] Cortes C Desler C Mazzoli A Chen JY Ferreira VP. The role of properdin and factor h in disease. Adv Immunol (2022) 153:1–90. doi: 10.1016/bs.ai.2021.12.001 35469595

[72] Masaki T Matsumoto M Yasuda R Levine RP Kitamura H Seya T. A covalent dimer of complement C4b serves as a subunit of a novel C5 convertase that involves no C3 derivatives. J Immunol (1991) 147:927–32. 1861081

[73] Polley MJ Nachman RL. Human platelet activation by C3a and C3a des-arg. J Exp Med (1983) 158:603–15. doi: 10.1084/jem.158.2.603 PMC2187348 6604123

[74] Propson NE Roy ER Litvinchuk A Kohl J Zheng H. Endothelial C3a receptor mediates vascular inflammation and blood-brain barrier permeability during aging. J Clin Invest (2021) 131(1):e140966. doi: 10.1172/JCI140966 PMC7773352 32990682

[75] Shivshankar P Li YD Mueller-Ortiz SL Wetsel RA. In response to complement anaphylatoxin peptides C3a and C5a, human vascular endothelial cells migrate and mediate the activation of b-cells and polarization of T-cells. FASEB J (2020) 34:7540–60. doi: 10.1096/fj.201902397R PMC11905332 32301538

[76] Laumonnier Y Karsten CM Kohl J. Novel insights into the expression pattern of anaphylatoxin receptors in mice and men. Mol Immunol (2017) 89:44–58. doi: 10.1016/j.molimm.2017.05.019 28600003

[77] Afzali B Noris M Lambrecht BN Kemper C. The state of complement in COVID-19. Nat Rev Immunol (2022) 2:77–84. doi: 10.1038/s41577-021-00665-1 PMC8672651 34912108

[78] Sendo F Yoshitake H Araki Y. Targeting of neutrophil activation in the early phase of the disease for prevention of coronavirus disease-19 severity. Microbiol Immunol (2022) 66:264–76. doi: 10.1111/1348-0421.12978 PMC9111295 35348252

[79] Shi H Zuo Y Navaz S Harbaugh A Hoy CK Gandhi AA. Endothelial cell-activating antibodies in COVID-19. Arthritis Rheumatol (2022) 74:1132–38. doi: 10.1002/art.42094 PMC9082472 35174669

[80] Carvelli J Demaria O Vely F Batista L Chouaki Benmansour N Fares J. Association of COVID-19 inflammation with activation of the C5a-C5aR1 axis. Nature (2020) 588:146–50. doi: 10.1038/s41586-020-2600-6 PMC7116884 32726800

[81] Fuchs TA Abed U Goosmann C Hurwitz R Schulze I Wahn V. Novel cell death program leads to neutrophil extracellular traps. J Cell Biol (2007) 176:231–41. doi: 10.1083/jcb.200606027 PMC2063942 17210947

[82] Mamtimin M Pinarci A Han C Braun A Anders HJ Gudermann T. Extracellular DNA traps: Origin, function and implications for anti-cancer therapies. Front Oncol (2022) 12:869706. doi: 10.3389/fonc.2022.869706 35574410 PMC9092261

[83] Martinod K Wagner DD. Thrombosis: tangled up in NETs. Blood (2014) 123:2768–76. doi: 10.1182/blood-2013-10-463646 PMC4007606 24366358

[84] Geddings JE Mackman N. New players in haemostasis and thrombosis. Thromb haemostasis (2014) 111:570–4. doi: 10.1160/TH13-10-0812 PMC4080798 24573314

[85] Loh JT Zhang B Teo JKH Lai RC Choo ABH Lam KP. Mechanism for the attenuation of neutrophil and complement hyperactivity by MSC exosomes. Cytotherapy (2022) 24:711–19. doi: 10.1016/j.jcyt.2021.12.003 PMC8843421 35177337

[86] Chen Y Li X Lin X Liang H Liu X Zhang X. Complement C5a induces the generation of neutrophil extracellular traps by inhibiting mitochondrial STAT3 to promote the development of arterial thrombosis. Thromb J (2022) 20:24. doi: 10.1186/s12959-022-00384-0 35488279 PMC9051782

[87] de Bont CM Boelens WC Pruijn GJM. NETosis, complement, and coagulation: a triangular relationship. Cell Mol Immunol (2019) 16:19–27. doi: 10.1038/s41423-018-0024-0 29572545 PMC6318284

[88] Middleton EA He XY Denorme F Campbell RA Ng D Salvatore SP. Neutrophil extracellular traps contribute to immunothrombosis in COVID-19 acute respiratory distress syndrome. Blood (2020) 136:1169–79. doi: 10.1182/blood.2020007008 PMC7472714 32597954

[89] Englert H Rangaswamy C Deppermann C Sperhake JP Krisp C Schreier D. Defective NET clearance contributes to sustained FXII activation in COVID-19-associated pulmonary thrombo-inflammation. EBioMedicine (2021) 67:103382. doi: 10.1016/j.ebiom.2021.103382 34000623 PMC8120108

[90] Skendros P Mitsios A Chrysanthopoulou A Mastellos DC Metallidis S Rafailidis P. Complement and tissue factor-enriched neutrophil extracellular traps are key drivers in COVID-19 immunothrombosis. J Clin Invest (2020) 130:6151–57. doi: 10.1172/JCI141374 PMC7598040 32759504

[91] Mastellos DC Pires BGP Fonseca BAL Fonseca NP Auxiliadora-martins M Mastaglio S. Complement C3 vs C5 inhibition in severe COVID-19: early clinical findings reveal differential biological efficacy. Clin Immunol (2020) 220:108598–8. doi: 10.1016/j.clim.2020.108598 PMC7501834 32961333

[92] Brinkmann V Reichard U Goosmann C Fauler B Uhlemann Y Weiss DS. Neutrophil extracellular traps kill bacteria. Science (2004) 303:1532–5. doi: 10.1126/science.1092385 15001782

[93] Shi Y Gauer JS Baker SR Philippou H Connell SD Ariens RAS. Neutrophils can promote clotting *via* FXI and impact clot structure *via* neutrophil extracellular traps in a distinctive manner in vitro. Sci Rep (2021) 11:1718. doi: 10.1038/s41598-021-81268-7 33462294 PMC7814028

[94] Keragala CB Draxler DF McQuilten ZK Medcalf RL. Haemostasis and innate immunity - a complementary relationship: A review of the intricate relationship between coagulation and complement pathways. Br J haematol (2018) 180:782–98. doi: 10.1111/bjh.15062 29265338

[95] Barranco-Medina S Pozzi N Vogt AD Di Cera E. Histone H4 promotes prothrombin autoactivation. J Biol Chem (2013) 288:35749–57. doi: 10.1074/jbc.M113.509786 PMC3861626 24178300

[96] Varju I Longstaff C Szabo L Farkas AZ Varga-Szabo VJ Tanka-Salamon A. DNA, Histones and neutrophil extracellular traps exert anti-fibrinolytic effects in a plasma environment. Thromb haemostasis (2015) 113:1289–98. doi: 10.1160/TH14-08-0669 25789443

[97] Ammollo CT Semeraro F Xu J Esmon NL Esmon CT. Extracellular histones increase plasma thrombin generation by impairing thrombomodulin-dependent protein c activation. J Thromb Haemost (2011) 9:1795–803. doi: 10.1111/j.1538-7836.2011.04422.x 21711444

[98] Xu J Zhang X Pelayo R Monestier M Ammollo CT Semeraro F. Extracellular histones are major mediators of death in sepsis. Nat Med (2009) 15:1318–21. doi: 10.1038/nm.2053 PMC2783754 19855397

[99] Longstaff C Varju I Sotonyi P Szabo L Krumrey M Hoell A. Mechanical stability and fibrinolytic resistance of clots containing fibrin, DNA, and histones. J Biol Chem (2013) 288:6946–56. doi: 10.1074/jbc.M112.404301 PMC3591605 23293023

[100] Glaser C Morser J Clarke J Blasko E McLean K Kuhn I. Oxidation of a specific methionine in thrombomodulin by activated neutrophil products blocks cofactor activity. J Clin Invest (1992) 90:2565–73. doi: 10.1172/JCI116151 PMC443416 1334978

[101] Higuchi DA Wun TC Likert KM Broze GJ. The effect of leukocyte elastase on tissue factor pathway inhibitor. Blood (1992) 79:1712–9. doi: 10.1182/blood.V79.7.1712.1712 1558967

[102] Maas C Renne T. Coagulation factor XII in thrombosis and inflammation. Blood (2018) 131:1903–9. doi: 10.1182/blood-2017-04-569111 29483100

[103] Srivastava P Gailani D. The rebirth of the contact pathway: a new therapeutic target. Curr Opin Hematol (2020) 27:311–9. doi: 10.1097/MOH.0000000000000603 PMC7596882 32740037

[104] Liu J Cooley BC Akinc A Butler J Borodovsky A. Knockdown of liver-derived factor XII by GalNAc-siRNA ALN-F12 prevents thrombosis in mice without impacting hemostatic function. Thromb Res (2020) 196:200–5. doi: 10.1016/j.thromres.2020.08.040 32896690

[105] Mailer RK Rangaswamy C Konrath S Emsley J Renne T. An update on factor XII-driven vascular inflammation. Biochim Biophys Acta Mol Cell Res (2022) 1869:119166. doi: 10.1016/j.bbamcr.2021.119166 34699874

[106] Craig T Magerl M Levy DS Reshef A Lumry WR Martinez-Saguer I. Prophylactic use of an anti-activated factor XII monoclonal antibody, garadacimab, for patients with C1-esterase inhibitor-deficient hereditary angioedema: a randomised, double-blind, placebo-controlled, phase 2 trial. Lancet (2022) 399:945–55. doi: 10.1016/S0140-6736(21)02225-X 35219377

[107] Shamanaev A Emsley J Gailani D. Proteolytic activity of contact factor zymogens. J Thromb Haemost (2021) 19:330–41. doi: 10.1111/jth.15149 PMC8552315 33107140

[108] Kannemeier C Shibamiya A Nakazawa F Trusheim H Ruppert C Markart P. Extracellular RNA constitutes a natural procoagulant cofactor in blood coagulation. Proc Natl Acad Sci United States America (2007) 104:6388–93. doi: 10.1073/pnas.0608647104 PMC1851071 17405864

[109] von Bruhl ML Stark K Steinhart A Chandraratne S Konrad I Lorenz M. Monocytes, neutrophils, and platelets cooperate to initiate and propagate venous thrombosis in mice in vivo. J Exp Med (2012) 209:819–35. doi: 10.1084/jem.20112322 PMC3328366 22451716

[110] Muller F Mutch NJ Schenk WA Smith SA Esterl L Spronk HM. Platelet polyphosphates are proinflammatory and procoagulant mediators in vivo. Cell (2009) 139:1143–56. doi: 10.1016/j.cell.2009.11.001 PMC2796262 20005807

[111] Renne T. The procoagulant and proinflammatory plasma contact system. Semin immunopathol (2012) 34:31–41. doi: 10.1007/s00281-011-0288-2 21858560

[112] Kohs TCL Lorentz CU Johnson J Puy C Olson SR Shatzel JJ. Development of coagulation factor XII antibodies for inhibiting vascular device-related thrombosis. Cell Mol Bioeng (2021) 14:161–75. doi: 10.1007/s12195-020-00657-6 PMC8010086 33868498

[113] Matafonov A Leung PY Gailani AE Grach SL Puy C Cheng Q. Factor XII inhibition reduces thrombus formation in a primate thrombosis model. Blood (2014) 123:1739–46. doi: 10.1182/blood-2013-04-499111 PMC3954054 24408325

[114] Demoulin S Godfroid E Hermans C. Dual inhibition of factor XIIa and factor XIa as a therapeutic approach for safe thromboprotection. J Thromb Haemost (2021) 19:323–9. doi: 10.1111/jth.15130 33047454

[115] Fang C Schmaier AH. Novel anti-thrombotic mechanisms mediated by mas receptor as result of balanced activities between the kallikrein/kinin and the renin-angiotensin systems. Pharmacol Res (2020) 160:105096. doi: 10.1016/j.phrs.2020.105096 32712319 PMC7378497

[116] Bekassy Z Lopatko Fagerstrom I Bader M Karpman D. Crosstalk between the renin-angiotensin, complement and kallikrein-kinin systems in inflammation. Nat Rev Immunol (2021) 22:411–28. doi: 10.1038/s41577-021-00634-8 PMC8579187 34759348

[117] Campbell WD Lazoura E Okada N Okada H. Inactivation of C3a and C5a octapeptides by carboxypeptidase r and carboxypeptidase n. Microbiol Immunol (2002) 46:131–4. doi: 10.1111/j.1348-0421.2002.tb02669.x 11939578

[118] Brown NJ Gainer JV Stein CM Vaughan DE. Bradykinin stimulates tissue plasminogen activator release in human vasculature. Hypertension (1999) 33:1431–5. doi: 10.1161/01.HYP.33.6.1431 10373228

[119] Simao F Ustunkaya T Clermont AC Feener EP. Plasma kallikrein mediates brain hemorrhage and edema caused by tissue plasminogen activator therapy in mice after stroke. Blood (2017) 129:2280–90. doi: 10.1182/blood-2016-09-740670 PMC5399481 28130211

[120] Ratnoff OD Pensky J Ogston D Naff GB. The inhibition of plasmin, plasma kallikrein, plasma permeability factor, and the C'1r subcomponent of the first component of complement by serum C'1 esterase inhibitor. J Exp Med (1969) 129:315–31. doi: 10.1084/jem.129.2.315 PMC2138601 4178758

[121] Howes JM Richardson VR Smith KA Schroeder V Somani R Shore A. Complement C3 is a novel plasma clot component with anti-fibrinolytic properties. Diabetes Vasc Dis Res (2012) 9:216–25. doi: 10.1177/1479164111432788 22253322

[122] Niculescu F Rus H. The role of complement activation in atherosclerosis. Immunologic Res (2004) 30:73–80. doi: 10.1385/IR:30:1:073 15258311

[123] Gushiken FC Han H Li J Rumbaut RE Afshar-Kharghan V. Abnormal platelet function in C3-deficient mice. J Thromb Haemost (2009) 7:865–70. doi: 10.1111/j.1538-7836.2009.03334.x PMC2867673 19291167

[124] Hamilton KK Hattori R Esmon CT Sims PJ. Complement proteins C5b-9 induce vesiculation of the endothelial plasma membrane and expose catalytic surface for assembly of the prothrombinase enzyme complex. J Biol Chem (1990) 265:3809–14. doi: 10.1016/S0021-9258(19)39666-8 2105954

[125] Wiedmer T Esmon CT Sims PJ. Complement proteins C5b-9 stimulate procoagulant activity through platelet prothrombinase. Blood (1986) 68:875–80. doi: 10.1182/blood.V68.4.875.875 3092889

[126] Hattori R Hamilton KK McEver RP Sims PJ. Complement proteins C5b-9 induce secretion of high molecular weight multimers of endothelial von willebrand factor and translocation of granule membrane protein GMP-140 to the cell surface. J Biol Chem (1989) 264:9053–60. doi: 10.1016/S0021-9258(18)81901-9 2470750

[127] Peerschke EI Yin W Ghebrehiwet B. Complement activation on platelets: implications for vascular inflammation and thrombosis. Mol Immunol (2010) 47:2170–5. doi: 10.1016/j.molimm.2010.05.009 PMC2904326 20621693

[128] Hisada Y Thalin C Lundstrom S Wallen H Mackman N. Comparison of microvesicle tissue factor activity in non-cancer severely ill patients and cancer patients. Thromb Res (2018) 165:1–5. doi: 10.1016/j.thromres.2018.03.001 29539580 PMC5943147

[129] Mazini L Rochette L Malka G. Exosomes contribution in COVID-19 patients' treatment. J Trans Med (2021) 19:234. doi: 10.1186/s12967-021-02884-5 PMC8165679 34059065

[130] Polley MJ Nachman RL. Human complement in thrombin-mediated platelet function: uptake of the C5b-9 complex. J Exp Med (1979) 150:633–45. doi: 10.1084/jem.150.3.633 PMC2185636 479764

[131] Krisinger MJ Goebeler V Lu Z Meixner SC Myles T Pryzdial EL. Thrombin generates previously unidentified C5 products that support the terminal complement activation pathway. Blood (2012) 120:1717–25. doi: 10.1182/blood-2012-02-412080 22802338

[132] Langer F Spath B Fischer C Stolz M Ayuk FA Kroger N. Rapid activation of monocyte tissue factor by antithymocyte globulin is dependent on complement and protein disulfide isomerase. Blood (2013) 121:2324–35. doi: 10.1182/blood-2012-10-460493 PMC3606067 23315166

[133] Podack ER Kolb WP Muller-Eberhard HJ. The SC5b-7 complex: formation, isolation, properties, and subunit composition. J Immunol (1977) 119:2024–9. 410885

[134] Falgarone G Chiocchia G. Chapter 8: Clusterin: A multifacet protein at the crossroad of inflammation and autoimmunity. Adv Cancer Res (2009) 104:139–70. doi: 10.1016/S0065-230X(09)04008-1 19878776

[135] Wat J Foley JH Krisinger MJ Ocariza LM Lei V Wasney G. Polyphosphate suppresses complement *via* the terminal pathway. Blood (2014) 123:768–76. doi: 10.1182/blood-2013-07-515726 PMC3907762 24335501

[136] Ninomiya H Sims PJ. The human complement regulatory protein CD59 binds to the alpha-chain of C8 and to the "b"domain of C9. J Biol Chem (1992) 267:13675–80. doi: 10.1016/S0021-9258(18)42266-1 1377690

[137] Wood AJT Vassallo A Summers C Chilvers ER Conway-Morris A. C5a anaphylatoxin and its role in critical illness-induced organ dysfunction. Eur J Clin Invest (2018) 48:e13028. doi: 10.1111/eci.13028 30229880

[138] Mizuno T Yoshioka K Mizuno M Shimizu M Nagano F Okuda T. Complement component 5 promotes lethal thrombosis. Sci Rep (2017) 7:42714. doi: 10.1038/srep42714 28205538 PMC5311936

[139] Hamad OA Nilsson PH Wouters D Lambris JD Ekdahl KN Nilsson B. Complement component C3 binds to activated normal platelets without preceding proteolytic activation and promotes binding to complement receptor 1. J Immunol (2010) 184:2686–92. doi: 10.4049/jimmunol.0902810 PMC2953618 20139276

[140] Martel C Cointe S Maurice P Matar S Ghitescu M Theroux P. Requirements for membrane attack complex formation and anaphylatoxins binding to collagen-activated platelets. PloS One (2011) 6:e18812. doi: 10.1371/journal.pone.0018812 21526204 PMC3078139

[141] Speth C Rambach G Wurzner R Lass-Florl C Kozarcanin H Hamad OA. Complement and platelets: Mutual interference in the immune network. Mol Immunol (2015) 67:108–18. doi: 10.1016/j.molimm.2015.03.244 25886718

[142] Nayak A Ferluga J Tsolaki AG Kishore U. The non-classical functions of the classical complement pathway recognition subcomponent C1q. Immunol Lett (2010) 131:139–50. doi: 10.1016/j.imlet.2010.03.012 20381531

[143] Yang J Furie BC Furie B. The biology of p-selectin glycoprotein ligand-1: its role as a selectin counterreceptor in leukocyte-endothelial and leukocyte-platelet interaction. Thromb Haemost (1999) 81:1–7. doi: 10.1055/s-0037-1614407 10348699

[144] Kozarcanin H Lood C Munthe-Fog L Sandholm K Hamad OA Bengtsson AA. The lectin complement pathway serine proteases (MASPs) represent a possible crossroad between the coagulation and complement systems in thromboinflammation. J Thromb Haemost (2016) 14:531–45. doi: 10.1111/jth.13208 26614707

[145] Eriksson O Chiu J Hogg PJ Atkinson JP Liszewski MK Flaumenhaft R. Thiol isomerase ERp57 targets and modulates the lectin pathway of complement activation. J Biol Chem (2019) 294:4878–88. doi: 10.1074/jbc.RA118.006792 PMC6442054 30670593

[146] Hamad OA Nilsson PH Lasaosa M Ricklin D Lambris JD Nilsson B. Contribution of chondroitin sulfate a to the binding of complement proteins to activated platelets. PloS One (2010) 5:e12889. doi: 10.1371/journal.pone.0012889 20886107 PMC2944812

[147] Wang H Ricklin D Lambris JD. Complement-activation fragment C4a mediates effector functions by binding as untethered agonist to protease-activated receptors 1 and 4. Proc Natl Acad Sci United States America (2017) 114:10948–53. doi: 10.1073/pnas.1707364114 PMC5642699 28973891

[148] Monsinjon T Gasque P Chan P Ischenko A Brady JJ Fontaine MC. Regulation by complement C3a and C5a anaphylatoxins of cytokine production in human umbilical vein endothelial cells. FASEB J (2003) 17:1003–14. doi: 10.1096/fj.02-0737com 12773483

[149] Foreman KE Vaporciyan AA Bonish BK Jones ML Johnson KJ Glovsky MM. C5a-induced expression of p-selectin in endothelial cells. J Clin Invest (1994) 94:1147–55. doi: 10.1172/JCI117430 PMC295185 7521884

[150] Fang W Guo ZH Zhang BQ Wu XF Li P Lv FL. [Effect of C5a on expression of thrombomodulin in endothelial cells *in vitro*]. Zhongguo wei zhong bing ji jiu yi xue = Chin Crit Care Med = Zhongguo weizhongbing jijiuyixue (2009) 21:168–71. 19278588

[151] Bongoni AK Lu B McRae JL Salvaris EJ Toonen EJM Vikstrom I. Complement-mediated damage to the glycocalyx plays a role in renal ischemia-reperfusion injury in mice. Transplant Direct (2019) 5:e341. doi: 10.1097/TXD.0000000000000881 30993186 PMC6445655

[152] Ueda Y Miwa T Ito D Kim H Sato S Gullipalli D. Differential contribution of C5aR and C5b-9 pathways to renal thrombic microangiopathy and macrovascular thrombosis in mice carrying an atypical hemolytic syndrome-related factor h mutation. Kidney Int (2019) 96:67–79. doi: 10.1016/j.kint.2019.01.009 30910380 PMC10084839

[153] Ritis K Doumas M Mastellos D Micheli A Giaglis S Magotti P. A novel C5a receptor-tissue factor cross-talk in neutrophils links innate immunity to coagulation pathways. J Immunol (2006) 177:4794–802. doi: 10.4049/jimmunol.177.7.4794 16982920

[154] Kim H Conway EM. Platelets and complement cross-talk in early atherogenesis. Front Cardiovasc Med (2019) 6:131. doi: 10.3389/fcvm.2019.00131 31555668 PMC6742699

[155] Khandelwal S Barnes A Rauova L Sarkar A Rux AH Yarovoi SV. Complement mediates binding and procoagulant effects of ultralarge HIT immune complexes. Blood (2021) 138:2106–16. doi: 10.1182/blood.2020009487 PMC8617432 34189574

[156] Al-Amer OM. The role of thrombin in haemostasis. Blood Coagul Fibrinolysis (2022) 33:145–48. doi: 10.1097/MBC.0000000000001130 35239615

[157] Huber-Lang M Sarma JV Zetoune FS Rittirsch D Neff TA McGuire SR. Generation of C5a in the absence of C3: a new complement activation pathway. Nat Med (2006) 12:682–7. doi: 10.1038/nm1419 16715088

[158] Amara U Flierl MA Rittirsch D Klos A Chen H Acker B. Molecular intercommunication between the complement and coagulation systems. J Immunol (2010) 185:5628–36. doi: 10.4049/jimmunol.0903678 PMC3123139 20870944

[159] Wetsel RA Kolb WP. Expression of C5a-like biological activities by the fifth component of human complement (C5) upon limited digestion with noncomplement enzymes without release of polypeptide fragments. J Exp Med (1983) 157:2029–48. doi: 10.1084/jem.157.6.2029 PMC2187036 6222137

[160] Keshari RS Silasi R Lupu C Taylor FB Jr. Lupu F. *In vivo*-generated thrombin and plasmin do not activate the complement system in baboons. Blood (2017) 130:2678–81. doi: 10.1182/blood-2017-06-788216 PMC5731087 29021229

[161] Mannes M Dopler A Zolk O Lang SJ Halbgebauer R Hochsmann B. Complement inhibition at the level of C3 or C5: mechanistic reasons for ongoing terminal pathway activity. Blood (2021) 137:443–55. doi: 10.1182/blood.2020005959 33507296

[162] Foley JH Walton BL Aleman MM O'Byrne AM Lei V Harrasser M. Complement activation in arterial and venous thrombosis is mediated by plasmin. EBioMedicine (2016) 5:175–82. doi: 10.1016/j.ebiom.2016.02.011 PMC4816834 27077125

[163] Nilsson PH Johnson C Quach QH Macpherson A Durrant O Pischke SE. A conformational change of complement C5 is required for thrombin-mediated cleavage, revealed by a novel ex vivo human whole blood model preserving full thrombin activity. J Immunol (2021) 207:1641–51. doi: 10.4049/jimmunol.2001471 PMC8428748 34380648

[164] Polley MJ Nachman R. The human complement system in thrombin-mediated platelet function. J Exp Med (1978) 147:1713–26. doi: 10.1084/jem.147.6.1713 PMC2184322 681879

[165] Del Conde I Cruz MA Zhang H Lopez JA Afshar-Kharghan V. Platelet activation leads to activation and propagation of the complement system. J Exp Med (2005) 201:871–9. doi: 10.1084/jem.20041497 PMC2213112 15781579

[166] Lidington EA Haskard DO Mason JC. Induction of decay-accelerating factor by thrombin through a protease-activated receptor 1 and protein kinase c-dependent pathway protects vascular endothelial cells from complement-mediated injury. Blood (2000) 96:2784–92. doi: 10.1182/blood.V96.8.2784 11023512

[167] Huber-Lang M Ekdahl KN Wiegner R Fromell K Nilsson B. Auxiliary activation of the complement system and its importance for the pathophysiology of clinical conditions. Semin immunopathol (2018) 40:87–102. doi: 10.1007/s00281-017-0646-9 28900700 PMC5794838

[168] Doring G. The role of neutrophil elastase in chronic inflammation. Am J Respir Crit Care Med (1994) 150:S114–7. doi: 10.1164/ajrccm/150.6_Pt_2.S114 7952645

[169] Huber-Lang M Denk S Fulda S Erler E Kalbitz M Weckbach S. Cathepsin d is released after severe tissue trauma *in vivo* and is capable of generating C5a in vitro. Mol Immunol (2012) 50:60–5. doi: 10.1016/j.molimm.2011.12.005 22244896

[170] DiScipio RG. The activation of the alternative pathway C3 convertase by human plasma kallikrein. Immunology (1982) 45:587–95. PMC1555245 6916710

[171] Saito A. Plasma kallikrein is activated on dermatan sulfate and cleaves factor h. Biochem Biophys Res Commun (2008) 370:646–50. doi: 10.1016/j.bbrc.2008.04.027 18413232

[172] Torzewski M Bhakdi S. Complement and atherosclerosis-united to the point of no return? Clin Biochem (2013) 46:20–5. doi: 10.1016/j.clinbiochem.2012.09.012 23010447

[173] Huang J Huffman JE Yamkauchi M Trompet S Asselbergs FW Sabater-Lleal M. Genome-wide association study for circulating tissue plasminogen activator levels and functional follow-up implicates endothelial STXBP5 and STX2. Arteriosclerosis thrombosis Vasc Biol (2014) 34:1093–101. doi: 10.1161/ATVBAHA.113.302088 PMC4009733 24578379

[174] Foley JH. Plasmin(ogen) at the nexus of fibrinolysis, inflammation, and complement. Semin Thromb hemostasis (2017) 43:135–42. doi: 10.1055/s-0036-1592302 28052305

[175] Madureira PA Surette AP Pipps KD Taboski MAS Miller VA Waisman DM. The role of annexin A2 heterotetramer in vascular fibrinolysis. Blood (2011) 118:4789–97. doi: 10.1182/blood-2011-06-334672 21908427

[176] Lighvani S Baik N Diggs JE Khaldoyanidi S Parmer RJ Miles LA. Regulation of macrophage migration by a novel plasminogen receptor plg-r _KT_ . Blood (2011) 118:5622–30. doi: 10.1182/blood-2011-03-344242 PMC3217361 21940822

[177] Heissig B Salama Y Takahashi S Osada T Hattori K. The multifaceted role of plasminogen in inflammation. Cell signalling (2020) 75:109761. doi: 10.1016/j.cellsig.2020.109761 32861744 PMC7452830

[178] Ward PA. A plasmin-split fragment of C'3 as a new chemotactic factor. J Exp Med (1967) 126:189–206. doi: 10.1084/jem.126.2.189 4226271 PMC2138318

[179] Barthel D Schindler S Zipfel PF. Plasminogen is a complement inhibitor. J Biol Chem (2012) 287:18831–42. doi: 10.1074/jbc.M111.323287 PMC3365705 22451663

[180] Rooijakkers SH van Wamel WJ Ruyken M van Kessel KP van Strijp JA. Anti-opsonic properties of staphylokinase. Microbes Infect (2005) 7:476–84. doi: 10.1016/j.micinf.2004.12.014 15792635

[181] Gasque P. Complement: a unique innate immune sensor for danger signals. Mol Immunol (2004) 41:1089–98. doi: 10.1016/j.molimm.2004.06.011 15476920

[182] Foley JH Peterson EA Lei V Wan LW Krisinger MJ Conway EM. Interplay between fibrinolysis and complement: Plasmin cleavage of iC3b modulates immune responses. J Thromb Haemost (2015) 13:610–8. doi: 10.1111/jth.12837 25556624

[183] Bumiller-Bini V de Freitas Oliveira-Tore C Carvalho TM Kretzschmar GC Goncalves LB Alencar NM. MASPs at the crossroad between the complement and the coagulation cascades - the case for COVID-19. Genet Mol Biol (2021) 44:e20200199. doi: 10.1590/1678-4685-gmb-2020-0199 33729332 PMC7982787

[184] Megyeri M Jani PK Kajdacsi E Dobo J Schwaner E Major B. Serum MASP-1 in complex with MBL activates endothelial cells. Mol Immunol (2014) 59:39–45. doi: 10.1016/j.molimm.2014.01.001 24472859

[185] Jani PK Kajdacsi E Megyeri M Dobo J Doleschall Z Futosi K. MASP-1 induces a unique cytokine pattern in endothelial cells: A novel link between complement system and neutrophil granulocytes. PloS One (2014) 9:e87104. doi: 10.1371/journal.pone.0087104 24489848 PMC3906121

[186] Jenny L Dobo J Gal P Schroeder V. MASP-1 of the complement system promotes clotting *via* prothrombin activation. Mol Immunol (2015) 65:398–405. doi: 10.1016/j.molimm.2015.02.014 25745807

[187] Krarup A Wallis R Presanis JS Gal P Sim RB. Simultaneous activation of complement and coagulation by MBL-associated serine protease 2. PloS One (2007) 2:e623. doi: 10.1371/journal.pone.0000623 17637839 PMC1910608

[188] Pagowska-Klimek I Cedzynski M. Mannan-binding lectin in cardiovascular disease. BioMed Res Int (2014) 2014:616817. doi: 10.1155/2014/616817 24877121 PMC4022110

[189] Ghebrehiwet B Randazzo BP Dunn JT Silverberg M Kaplan AP. Mechanisms of activation of the classical pathway of complement by hageman factor fragment. J Clin Invest (1983) 71:1450–6. doi: 10.1172/JCI110898 PMC437009 6304147

[190] La Bonte LR Pavlov VI Tan YS Takahashi K Takahashi M Banda NK. Mannose-binding lectin-associated serine protease-1 is a significant contributor to coagulation in a murine model of occlusive thrombosis. J Immunol (2012) 188:885–91. doi: 10.4049/jimmunol.1102916 PMC3253146 22156595

[191] Liang RA Hoiland II T. Ueland P Snir O Hindberg K Braekkan SK. Plasma levels of mannose-binding lectin and future risk of venous thromboembolism. J Thromb Haemost (2019) 17:1661–9. doi: 10.1111/jth.14539 31220397

[192] Flude BM Nannetti G Mitchell P Compton N Richards C Heurich M. Targeting the complement serine protease MASP-2 as a therapeutic strategy for coronavirus infections. Viruses (2021) 13(2):312. doi: 10.3390/v13020312 33671334 PMC7923061

[193] Gavriilaki E Peffault de Latour R Risitano AM. Advancing therapeutic complement inhibition in hematologic diseases: PNH and beyond. Blood (2021) 139:3571–82. doi: 10.1182/blood.2021012860 34482398

[194] Huntington JA. Serpin structure, function and dysfunction. J Thromb Haemostasis (2011) 9:26–34. doi: 10.1111/j.1538-7836.2011.04360.x 21781239

[195] Ellis V Scully M MacGregor I Kakkar V. Inhibition of human factor xa by various plasma protease inhibitors. Biochim Biophys Acta (1982) 701:24–31. doi: 10.1016/0167-4838(82)90307-7 6173074

[196] Osterud B Miller-Andersson M Abildgaard U Prydz H. The effect of antithrombin III on the activity of the coagulation factors VII, IX and X. Thromb Haemost (1976) 35:295–304. doi: 10.1055/s-0038-1647922 61631

[197] Patnaik MM Moll S. Inherited antithrombin deficiency: a review. Haemophilia (2008) 14:1229–39. doi: 10.1111/j.1365-2516.2008.01830.x 19141163

[198] Parej K Dobo J Zavodszky P Gal P. The control of the complement lectin pathway activation revisited: both C1-inhibitor and antithrombin are likely physiological inhibitors, while alpha2-macroglobulin is not. Mol Immunol (2013) 54:415–22. doi: 10.1016/j.molimm.2013.01.009 23399388

[199] Zeerleder S. C1-inhibitor: more than a serine protease inhibitor. Semin Thromb hemostasis (2011) 37:362–74. doi: 10.1055/s-0031-1276585 21805442

[200] Kerr FK Thomas AR Wijeyewickrema LC Whisstock JC Boyd SE Kaiserman D. Elucidation of the substrate specificity of the MASP-2 protease of the lectin complement pathway and identification of the enzyme as a major physiological target of the serpin, C1-inhibitor. Mol Immunol (2008) 45:670–7. doi: 10.1016/j.molimm.2007.07.008 17709141

[201] Wuillemin WA Minnema M Meijers JC Roem D Eerenberg AJ Nuijens JH. Inactivation of factor XIa in human plasma assessed by measuring factor XIa-protease inhibitor complexes: major role for C1-inhibitor. Blood (1995) 85:1517–26. doi: 10.1182/blood.V85.6.1517.bloodjournal8561517 7534133

[202] Levi M Roem D Kamp AM de Boer JP Hack CE ten Cate JW. Assessment of the relative contribution of different protease inhibitors to the inhibition of plasmin in vivo. Thromb haemostasis (1993) 69:141–6. doi: 10.1055/s-0038-1651570 7681223

[203] Ziccardi RJ. Activation of the early components of the classical complement pathway under physiologic conditions. J Immunol (1981) 126:1769–73. 7217665

[204] Rossi V Cseh S Bally I Thielens NM Jensenius JC Arlaud GJ. Substrate specificities of recombinant mannan-binding lectin-associated serine proteases-1 and -2. J Biol Chem (2001) 276:40880–7. doi: 10.1074/jbc.M105934200 11527969

[205] Pike RN Wijeyewickrema LC. The molecular switches controlling the interaction between complement proteases of the classical and lectin pathways and their substrates. Curr Opin Struct Biol (2013) 23:820–7. doi: 10.1016/j.sbi.2013.07.016 23932199

[206] Wijeyewickrema LC Lameignere E Hor L Duncan RC Shiba T Travers RJ. Polyphosphate is a novel cofactor for regulation of complement by a serpin, C1 inhibitor. Blood (2016) 128:1766–76. doi: 10.1182/blood-2016-02-699561 PMC5043130 27338096

[207] Beinrohr L Murray-Rust TA Dyksterhuis L Zavodszky P Gal P Pike RN. Serpins and the complement system. Methods enzymol (2011) 499:55–75. doi: 10.1016/B978-0-12-386471-0.00004-3 21683249

[208] Malik RA Zhou J Fredenburgh JC Truong TK Crosby JR Revenko AS. Polyphosphate-induced thrombosis in mice is factor XII dependent and is attenuated by histidine-rich glycoprotein. Blood Adv (2021) 5:3540–51. doi: 10.1182/bloodadvances.2021004567 PMC8945577 34474475

[209] Conway EM. Polyphosphates and complement activation. Front Med (2019) 6:67. doi: 10.3389/fmed.2019.00067 PMC6458250 31019911

[210] Rangaswamy C Englert H Deppermann C Renne T. Polyanions in coagulation and thrombosis: Focus on polyphosphate and neutrophils extracellular traps. Thromb haemostasis (2021) 121:1021–30. doi: 10.1055/a-1336-0526 33307564

[211] Jiang H Wagner E Zhang H Frank MM. Complement 1 inhibitor is a regulator of the alternative complement pathway. J Exp Med (2001) 194:1609–16. doi: 10.1084/jem.194.11.1609 PMC2193533 11733575

[212] Levi M Cohn DM Zeerleder S. Hereditary angioedema: Linking complement regulation to the coagulation system. Res Pract Thromb Haemost (2019) 3:38–43. doi: 10.1002/rth2.12175 30656274 PMC6332742

[213] Sundler Bjorkman L Persson B Aronsson D Skattum L Nordenfelt P Egesten A. Comorbidities in hereditary angioedema-a population-based cohort study. Clin Transl Allergy (2022) 12:e12135. 35344299 10.1002/clt2.12135 PMC8967273

[214] Mast AE. Tissue factor pathway inhibitor: Multiple anticoagulant activities for a single protein. Arterioscler Thromb Vasc Biol (2016) 36:9–14. doi: 10.1161/ATVBAHA.115.305996 26603155 PMC4690769

[215] Maroney SA Ellery PE Mast AE. Alternatively spliced isoforms of tissue factor pathway inhibitor. Thromb Res (2010) 125 Suppl 1:S52–6. doi: 10.1016/j.thromres.2010.01.038 PMC2839007 20176395

[216] Ellery PE Adams MJ. Tissue factor pathway inhibitor: then and now. Semin Thromb hemostasis (2014) 40:881–6. doi: 10.1055/s-0034-1395153 25377319

[217] Keizer MP Pouw RB Kamp AM Patiwael S Marsman G Hart MH. TFPI inhibits lectin pathway of complement activation by direct interaction with MASP-2. Eur J Immunol (2015) 45:544–50. doi: 10.1002/eji.201445070 25359215

[218] Skjoedt MO Hummelshoj T Palarasah Y Honore C Koch C Skjodt K. A novel mannose-binding lectin/ficolin-associated protein is highly expressed in heart and skeletal muscle tissues and inhibits complement activation. J Biol Chem (2010) 285:8234–43. doi: 10.1074/jbc.M109.065805 PMC2832975 20053996

[219] Holmquist E Okroj M Nodin B Jirstrom K Blom AM. Sushi domain-containing protein 4 (SUSD4) inhibits complement by disrupting the formation of the classical C3 convertase. FASEB J (2013) 27:2355–66. doi: 10.1096/fj.12-222042 23482636

[220] Rawal N Pangburn MK. C5 convertase of the alternative pathway of complement. kinetic analysis of the free and surface-bound forms of the enzyme. J Biol Chem (1998) 273:16828–35. doi: 10.1074/jbc.273.27.16828 9642242

[221] Rawal N Pangburn MK. Formation of high affinity C5 convertase of the classical pathway of complement. J Biol Chem (2003) 278:38476–83. doi: 10.1074/jbc.M307017200 12878586

[222] Pangburn MK Muller-Eberhard HJ. The alternative pathway of complement. Springer Semin immunopathol (1984) 7:163–92. doi: 10.1007/BF01893019 6238433

[223] Licht C Pluthero FG Li L Christensen H Habbig S Hoppe B. Platelet-associated complement factor h in healthy persons and patients with atypical HUS. Blood (2009) 114:4538–45. doi: 10.1182/blood-2009-03-205096 19704120

[224] Clark SJ Ridge LA Herbert AP Hakobyan S Mulloy B Lennon R. Tissue-specific host recognition by complement factor h is mediated by differential activities of its glycosaminoglycan-binding regions. J Immunol (2013) 190:2049–57. doi: 10.4049/jimmunol.1201751 PMC3672945 23365078

[225] Kim DD Song WC. Membrane complement regulatory proteins. Clin Immunol (2006) 118:127–36. doi: 10.1016/j.clim.2005.10.014 16338172

[226] Ekdahl KN Nilsson B. Phosphorylation of complement component C3 and C3 fragments by a human platelet protein kinase. inhibition of factor I-mediated cleavage of C3b. J Immunol (1995) 154:6502–10. 7539023

[227] Nilsson-Ekdahl K Nilsson B. Phosphorylation of C3 by a casein kinase released from activated human platelets increases opsonization of immune complexes and binding to complement receptor type 1. Eur J Immunol (2001) 31:1047–54. doi: 10.1002/1521-4141(200104)31:4<1047::AID-IMMU1047>3.0.CO;2-Y 11298329

[228] Puy C Pang J Reitsma SE Lorentz CU Tucker EI Gailani D. Cross-talk between the complement pathway and the contact activation system of coagulation: Activated factor XI neutralizes complement factor h. J Immunol (2021) 206:1784–92. doi: 10.4049/jimmunol.2000398 PMC8030746 33811105

[229] Chen LJ Liu DT Tam PO Chan WM Liu K Chong KK. Association of complement factor h polymorphisms with exudative age-related macular degeneration. Mol Vision (2006) 12:1536–42. 17167412

[230] Thangaraj SS Christiansen SH Graversen JH Sidelmann JJ Hansen SWK Bygum A. Contact activation-induced complex formation between complement factor h and coagulation factor XIIa. J Thromb Haemost (2020) 18:876–84. doi: 10.1111/jth.14742 31984663

[231] Ferreira VP Herbert AP Cortes C McKee KA Blaum BS Esswein ST. The binding of factor h to a complex of physiological polyanions and C3b on cells is impaired in atypical hemolytic uremic syndrome. J Immunol (2009) 182:7009–18. doi: 10.4049/jimmunol.0804031 PMC2696619 19454698

[232] Saunders RE Abarrategui-Garrido C Fremeaux-Bacchi V Goicoechea de Jorge E Goodship TH Lopez Trascasa M. The interactive factor h-atypical hemolytic uremic syndrome mutation database and website: update and integration of membrane cofactor protein and factor I mutations with structural models. Hum Mutat (2007) 28:222–34. doi: 10.1002/humu.20435 17089378

[233] Nester CM Barbour T de Cordoba SR Dragon-Durey MA Fremeaux-Bacchi V Goodship TH. Atypical aHUS: State of the art. Mol Immunol (2015) 67:31–42. doi: 10.1016/j.molimm.2015.03.246 25843230

[234] Tsai HM. A mechanistic approach to the diagnosis and management of atypical hemolytic uremic syndrome. Transfusion Med Rev (2014) 28:187–97. doi: 10.1016/j.tmrv.2014.08.004 25280590

[235] Mele C Remuzzi G Noris M. Hemolytic uremic syndrome. Semin immunopathol (2014) 36:399–420. doi: 10.1007/s00281-014-0416-x 24526222

[236] Bu F Maga T Meyer NC Wang K Thomas CP Nester CM. Comprehensive genetic analysis of complement and coagulation genes in atypical hemolytic uremic syndrome. J Am Soc Nephrol (2014) 25:55–64. doi: 10.1681/ASN.2013050453 24029428 PMC3871781

[237] Esmon CT Stenflo J Suttie JW. A new vitamin K-dependent protein. a phospholipid-binding zymogen of a serine esterase. J Biol Chem (1976) 251:3052–6. doi: 10.1016/S0021-9258(17)33498-1 1270437

[238] Griffin JH Evatt B Zimmerman TS Kleiss AJ Wideman C. Deficiency of protein c in congenital thrombotic disease. J Clin Invest (1981) 68:1370–3. doi: 10.1172/JCI110385 PMC370934 6895379

[239] Bajzar L Manuel R Nesheim M. Purification and characterization of TAFI, a thrombin-activatable fibrinolysis inhibitor. J Biol Chem (1995) 270:14477–84. doi: 10.1074/jbc.270.24.14477 7782309

[240] Bellac CL Dufour A Krisinger MJ Loonchanta A Starr AE Auf dem Keller U. Macrophage matrix metalloproteinase-12 dampens inflammation and neutrophil influx in arthritis. Cell Rep (2014) 9:618–32. doi: 10.1016/j.celrep.2014.09.006 25310974

[241] Delvaeye M Noris M De Vriese A Esmon CT Esmon NL Ferrell G. Thrombomodulin mutations in atypical hemolytic-uremic syndrome. New Engl J Med (2009) 361:345–57. doi: 10.1056/NEJMoa0810739 PMC3530919 19625716

[242] Heurich M Preston RJ O'Donnell VB Morgan BP Collins PW. Thrombomodulin enhances complement regulation through strong affinity interactions with factor h and C3b-factor h complex. Thromb Res (2016) 145:84–92. doi: 10.1016/j.thromres.2016.07.017 27513882

[243] Tateishi K Imaoka M Matsushita M. Dual modulating functions of thrombomodulin in the alternative complement pathway. Biosci Trends (2016) 10:231–4. doi: 10.5582/bst.2016.01052 27210597

[244] Loghmani H Conway EM. Exploring traditional and non-traditional roles for thrombomodulin. Blood (2018) 132:148–58. doi: 10.1182/blood-2017-12-768994 29866818

[245] Zheng XL Sadler JE. Pathogenesis of thrombotic microangiopathies. Annu Rev Pathol (2008) 3:249–77. doi: 10.1146/annurev.pathmechdis.3.121806.154311 PMC2582586 18215115

[246] Turner NA Moake J. Assembly and activation of alternative complement components on endothelial cell-anchored ultra-large von willebrand factor links complement and hemostasis-thrombosis. PloS One (2013) 8:e59372. doi: 10.1371/journal.pone.0059372 23555663 PMC3612042

[247] Feng S Liang X Cruz MA Vu H Zhou Z Pemmaraju N. The interaction between factor h and Von willebrand factor. PloS One (2013) 8:e73715. doi: 10.1371/journal.pone.0073715 23991205 PMC3753316

[248] Rayes J Roumenina LT Dimitrov JD Repesse Y Ing M Christophe O. The interaction between factor h and VWF increases factor h cofactor activity and regulates VWF prothrombotic status. Blood (2014) 123:121–5. doi: 10.1182/blood-2013-04-495853 24014239

[249] Nolasco L Nolasco J Feng S Afshar-Kharghan V Moake J. Human complement factor h is a reductase for large soluble von willebrand factor multimers–brief report. Arteriosclerosis thrombosis Vasc Biol (2013) 33:2524–8. doi: 10.1161/ATVBAHA.113.302280 24008159

[250] Turner N Nolasco L Nolasco J Sartain S Moake J. Thrombotic microangiopathies and the linkage between von willebrand factor and the alternative complement pathway. Semin Thromb hemostasis (2014) 40:544–50. doi: 10.1055/s-0034-1383547 24967890

[251] Feng S Liang X Kroll MH Chung DW Afshar-Kharghan V. Von willebrand factor is a cofactor in complement regulation. Blood (2015) 125:1034–7. doi: 10.1182/blood-2014-06-585430 PMC4319234 25395424

[252] Schmidt CQ Schrezenmeier H Kavanagh D. Complement and the prothrombotic state. Blood (2021) 139:1954–72. doi: 10.1182/blood.2020007206 34415298

[253] Chamardani TM Amiritavassoli S. Inhibition of NETosis for treatment purposes: friend or foe? Mol Cell Biochem (2022) 477:673–88. doi: 10.1007/s11010-021-04315-x PMC8736330 34993747

